# Implementing the INTERGROWTH-21^st^ gestational dating and fetal and newborn growth standards in peri-urban Nairobi, Kenya: Provider experiences, uptake and clinical decision-making

**DOI:** 10.1371/journal.pone.0213388

**Published:** 2019-03-08

**Authors:** Linda Vesel, Kojo Nimako, Rachel M. Jones, Meghan Munson, Sarah Little, Henry Njogu, Irene Njuru, Teresa Ogolla, Grace Kimenju, Mary Nell Wegner, Sathyanath Rajasekharan, Nicholas Pearson, Ana Langer

**Affiliations:** 1 Department of Global Health and Population, Harvard T.H. Chan School of Public Health, Boston, Massachusetts, United States of America; 2 Jacaranda Health, Nairobi, Kenya; Monash University, AUSTRALIA

## Abstract

**Background:**

Perinatal and newborn complications are major risk factors for unfavorable fetal and neonatal outcomes. Gestational dating and growth monitoring can be instrumental in the identification and management of high-risk pregnancies and births. The INTERGROWTH-21^st^ Project developed the first global standards for gestational dating and fetal and newborn growth monitoring, supplying a toolkit for clinicians. This study aimed to assess the feasibility and acceptability of the first known implementation study of these standards in a low resource setting.

**Methods:**

The study was performed in two 12-month phases from March 2016 to March 2018 at Jacaranda Health, a private maternity hospital in peri-urban Nairobi, Kenya. In-depth interviews, focus group discussions and a provider survey were utilized to evaluate providers’ experiences during implementation. Client chart data, for pregnant women attending antenatal care and/or delivering at Jacaranda Health along with their newborns, were captured to assess uptake and effect of the standards on clinical decision-making.

**Results:**

Facility-level support and provider buy-in proved to be critical factors driving the success of implementing the standards. However, additional support was needed to strengthen capacity to conduct and interpret ultrasounds and maintain motivation among providers. We observed a significant increase in the uptake of obstetric ultrasounds, particularly gestational dating, during the implementation of the standards. Although no significant changes were detected in the identification of high-risk pregnancies, referrals and deliveries by Cesarean section during implementation, we did observe a significant reduction in inductions for post-date. No significant barriers were reported regarding the use of the newborn standards. Over 80% of providers advocated for the standards to remain in place with some enhancements related mainly to training, advocacy and procurement.

**Conclusions:**

The findings are timely with increasing global adoption of the standards and the challenging and multi-faceted nature of translating new, evidence-based guidelines into routine clinical practice.

## Introduction

Despite progress made in reducing maternal and newborn mortality, large gaps remain. Currently, 2.6 million neonates die yearly worldwide, most from preventable causes [1]. Fetal growth restriction, small-for-gestational age (SGA) and preterm birth (<37 weeks of gestation) are major risk factors for unfavorable fetal and neonatal outcomes; preterm birth complications remain the leading cause of death in the first month of life [2–5]. Many of these deaths can be prevented through quality antenatal and delivery care, namely the identification and management of high-risk pregnancies and births. Key components of this care include the use of standard ultrasound algorithms to date pregnancies and monitor fetal growth, newborn anthropometry to assess size at birth, the support of a trained workforce for early identification of complications and timely referral for advanced care [3, 6, 7]. Ultrasound guided assessment, used in combination with an indication of the last menstrual period (LMP), is the gold standard measurement for gestational age early in pregnancy; it has been shown to increase the accuracy of pregnancy dating and, consequently, influence outcomes [5, 8, 9]. Newborn anthropometry, based on an international standard of optimal growth, is instrumental in identifying SGA and low birth weight newborns (<2.5kg) and referring them for advanced care [10]. However, utilization of growth standards requires supply and demand side inputs including training of staff, procurement of equipment, changes in community perceptions for care-seeking and early presentation for antenatal care (ANC) in the case of ultrasound standards [5].

The INTERGROWTH-21^st^ Project developed the first global standards for gestational dating, fetal growth monitoring, and newborn size at birth, supplying a toolkit for clinicians working to reduce the burden of preterm birth as well as growth faltering and its complications [11]. These prescriptive standards are based on a global population of healthy women experiencing proper nutrition and minimal exposure to environmental risk factors for poor fetal growth sampled from eight countries around the world [11]. As a result, they address the World Health Organization (WHO) recommendation to develop and implement standards describing how fetuses and children should grow [11]. Recognizing that the translation of new guidelines to clinical practice can be challenging and multi-faceted, particularly in a low-resource setting, this paper documents the first known implementation study of the INTERGROWTH-21^st^ standards in routine clinical practice [12]. Our study evaluated the feasibility and acceptability of implementing the INTERGROWTH-21^st^ standards in a health facility in peri-urban Nairobi, Kenya and highlights key considerations for their integration into clinical practice in a low-resource setting. The primary objective was to assess providers’ perceptions of the facilitators and barriers to the use of the standards. The secondary objective was to evaluate the association between the implementation of the standards and clinical decision-making outcomes.

## Methods

### Study design & setting

This implementation research study was performed at Jacaranda Health, a private maternity hospital in peri-urban Nairobi. The health facility was chosen based on its mandate to provide high quality, low-cost ANC, delivery, family planning and child welfare services to the surrounding community and serve as a model for private and public facilities in Kenya. The study was conducted over a period of two years (March 2016 to March 2018), consisting of a 12-month pre-implementation phase which included training, adaptations to protocols and baseline data collection, and a 12-month implementation phase which included the integration of the INTERGROWTH-21^st^ standards into clinical workflow and data collection.

The INTERGROWTH-21^st^ standards provide new guidelines for gestational dating and fetal growth monitoring using ultrasounds. They also include newborn size at birth charts to plot growth percentiles based on gestational age at birth and sex, as well as newborn weight, length and head circumference in order to identify an accurate newborn diagnosis (small, appropriate or large for gestational age) [11]. Gestational dating ultrasounds were offered free of charge during the implementation phase to facilitate compliance. As part of the study, new equipment, including a digital scale with an attached measuring rod and disposable head circumference measuring tapes, were supplied to the facility. Jacaranda Health’s clinical protocols on ANC, high-risk pregnancy identification, referrals, gestational dating, fetal growth monitoring and newborn anthropometry were updated to match the new WHO guidelines and support INTERGROWTH-21^st^ protocols [13, 14]. Finally, new fields were introduced into clinical charting forms to facilitate the evaluation of the standards. In the first six months of the implementation phase, three trained and certified nurse-midwives performed the gestational dating ultrasounds as part of their regular ANC duties. After a mid-implementation review, the ultrasound delivery model was changed to have one nurse-midwife dedicated exclusively to the provision of gestational dating ultrasounds three days a week. All fetal growth monitoring scans were performed by the resident expert sonographer. Further details of the study setting, design and description can be found in the study protocol paper [15].

### Study participants

The study population included pregnant women attending ANC and/or delivering at Jacaranda Health as well as their newborns. The eligibility criteria varied for the three standards included in the implementation package—gestational dating, fetal growth monitoring and newborn size at birth. Pregnant women with a viable fetus presenting for their first ANC visit between eight and 26 weeks of gestation were eligible for a free scan using the gestational dating standards. Pregnant women with a viable fetus presenting for an ANC visit after 26 weeks of gestation and classified as high-risk based on their clinical history or presentation were eligible for an ultrasound using the fetal growth standards. Finally, all newborns who were delivered at Jacaranda Health qualified for newborn anthropometry using the newborn size at birth standards. Providers who were trained on the purpose and use of the INTERGROWTH-21^st^ standards and who delivered ANC and/or newborn anthropometry services at Jacaranda Health were eligible for participation in the in-depth interviews (IDIs), focus group discussions (FGDs) and the provider survey. Cadres of Jacaranda Health staff involved in the study included physicians, clinic managers, sonographers and nurse-midwives.

### Data collection

We utilized both quantitative and qualitative data collection tools. Providers’ experiences implementing the standards and the associated facilitators and barriers were examined through IDIs, FGDs and a short attitudes and satisfaction survey (S1, S2 and S3 Files). IDIs and FGDs were conducted by two experienced research assistants using piloted, semi-structured guides soliciting providers’ insights and perceptions of the training, implementation process, and effect of the standards on workload, workflow and accuracy of diagnoses. Interviews and discussions were administered once during the pre-implementation phase and twice during the implementation phase (at four and 12 months). A total of 19, 18 and 16 providers were either interviewed or participated in an FGD in the three data collection periods respectively (Table 1). The provider survey was administered to 29 clinicians at Jacaranda Health by a research assistant via SurveyMonkey [16] during the implementation phase (at eight months).

**Table 1:** Qualitative data collection.

Type of Provider	Timeframe & Data Collection Mode					
	Pre-Implementation Phase		Implementation Phase 4-Months		Implementation Phase 12-Months	
	In-depth Interviews	Focus Group Discussions	In-depth Interviews	Focus Group Discussions	In-depth Interviews	Focus Group Discussions
Clinic Manager	2		2	-	2	-
Nurse-Midwife (Newborn Anthropometry)	2	2 (5, 5)	-	3 (5, 4, 3)	10	-
Nurse-Midwife (Ultrasound)	4	-	3	-	4	-
Physician	1	-	1	-	-	-
TOTAL	9	10	6	12	16	-
	19		18		16	

For in-depth interviews: numbers in cells indicate individuals who were interviewed.

For focus group discussions: numbers in cells outside the parentheses indicate the number of focus groups conducted while the numbers in the parentheses separated by commas indicate the number of participants in each focus group.

The following outcomes were assessed using clinical data captured through client charts and the facility’s external referral logbook (S4 File) for 12-months before and 12-months during the implementation of the standards: uptake of gestational dating ultrasounds, fetal growth monitoring ultrasounds and newborn anthropometry; proportion of pregnant women identified as high-risk who were referred internally or externally; proportion of pregnant women undergoing a gestational dating scan whose labor was induced on account of post-date pregnancies; and proportion of pregnant women undertaking a gestational dating scan who delivered via Cesarean section. All client chart data were entered and managed in REDCap^®^, an electronic data capture system [17].

The quality of ultrasounds performed as part of the implementation of the standards was assessed by the quality assurance team of the original INTERGROWTH-21st Project at Oxford University. A total of 580 images capturing one of four fetal measurements—crown-rump length, head circumference, femur length and biparietal diameter—on three different ultrasound machines were reviewed by an expert sonographer at Oxford University in seven batches throughout the implementation phase. Seventy percent of the ultrasounds that yielded these images were performed by the nurse-midwife responsible for conducting all gestational dating scans during the second half of the implementation phase. The biparietal diameter was a measurement done routinely only by the expert sonographer as part of fetal growth monitoring scans. Quality was measured on a scale from zero to four or six (depending on the measurement) and was based on the fulfillment of specific criteria detailed in the INTERGROWTH-21^st^ protocols [14].

### Data analysis

All IDI and FGD transcripts were transcribed by an independent transcriber and de-identified before analysis was conducted. Transcripts were double-coded by two independent analysts to draw out themes using NVivo 11 software [18] at each data collection time point; results were compared across time when relevant. Provider demographic data were derived from records kept by Jacaranda Health’s Human Resources Department. Provider survey results were exported from SurveyMonkey [16] and analyzed in Microsoft Excel.

The quantitative data were analyzed using the Stata Statistical Software Package, Version 15 [19]. Descriptive analyses were performed on the data collected. Frequencies and percentages were defined for the categorical variables of interest in the two study phases and means (or medians) were derived for the continuous variables. For categorical variables, which formed the bulk of the data, differences between the two phases were assessed using chi-square analyses. For the few continuous variables, an analysis of variance (ANOVA) was performed. Statistical significance for all analyses was set at a 95% confidence limit (α = 0.05, with a Bonferroni correction in cases of multiple pairwise comparisons).

### Ethical considerations

Ethical clearance for the study was obtained from the Amref Ethics and Scientific Review Committee in Kenya and the Harvard T.H. Chan School of Public Health Institutional Review Board in the United States. Facility-level written informed consent was obtained from Jacaranda Health management to use routine client chart data emphasizing that patient identifiable information would not be analyzed or disseminated at any point. All clinicians and clients who participated in IDIs and FGDs were recruited, enrolled and provided informed consent via a signature.

## Results

### Demographics

Thirty-two providers were involved in data collection via IDIs, FGDs or the survey. Nearly 75% of these providers were female with an average age of 34 years. The majority of providers (72%) had a Diploma in Nursing (three-year professional course), 22% had a Bachelors in Nursing (four-year professional degree) and 6% had a medical degree with a specialization in obstetrics and gynecology. Most of these providers (88%) worked full-time at Jacaranda Health and, on average, had been working there for three years; around 20% of providers joined after the start of the implementation phase in March 2017 and 13% left before implementation came to an end in early March 2018.

**Fig 1:**
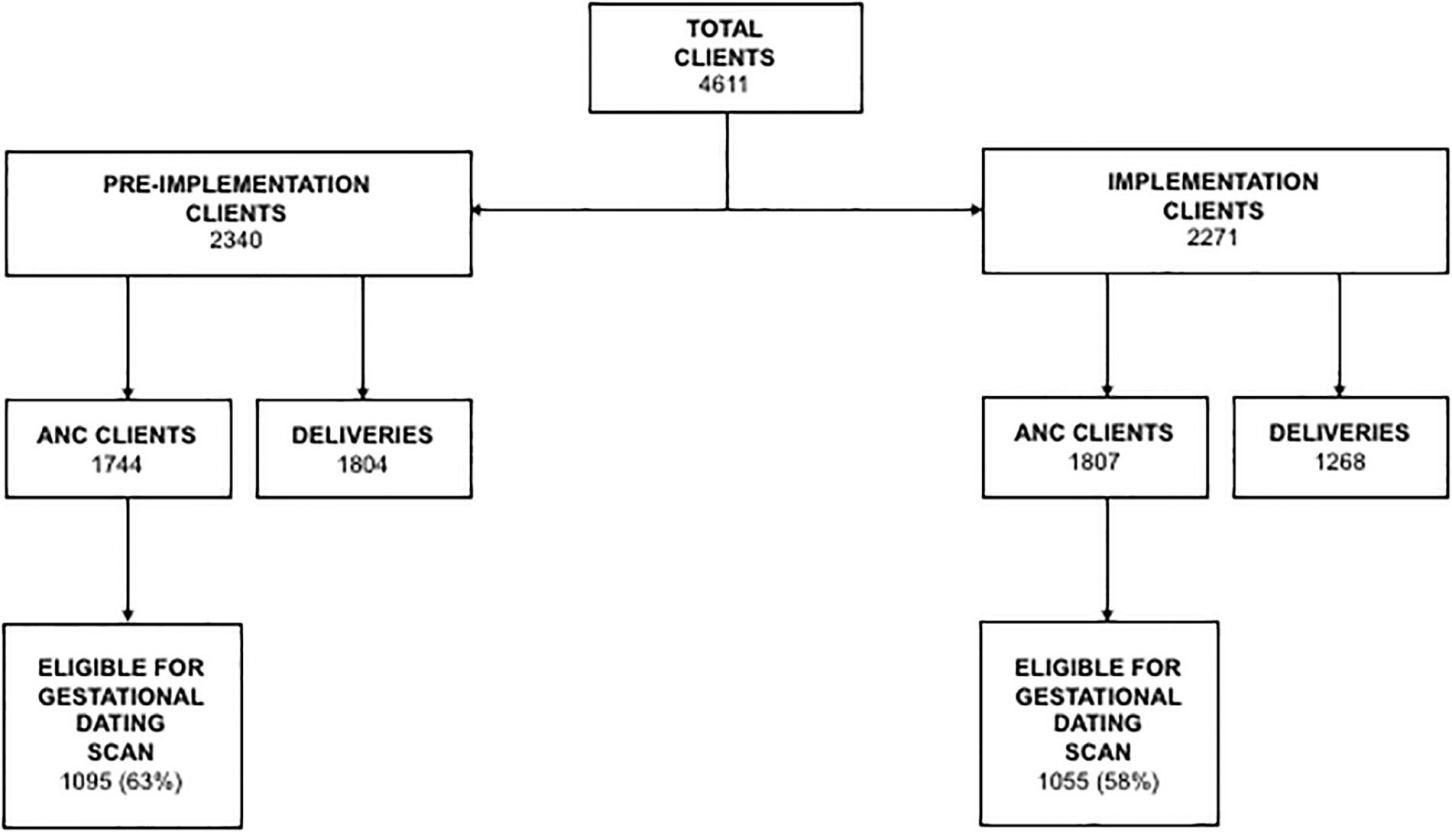
Client flowchart.

Clinical data were collected for 4611 clients attending ANC and/or delivering at Jacaranda Health over two years (Fig 1). During the pre-implementation phase, data were captured for 2340 clients of which 1744 attended ANC at Jacaranda Health. There were a total of 1084 deliveries during this period, with 495 to clients also attending ANC at Jacaranda Health and 589 to delivery-only walk-ins. During the implementation phase, charts were reviewed for 2271 clients of whom 1807 attended ANC at Jacaranda Health. There were 1268 deliveries during this period, with 587 to clients who also attended ANC during the implementation phase, 218 to those who attended ANC during the pre-implementation phase and 463 to delivery-only walk-ins. The age distribution of clients in the pre-implementation and implementation phases was similar, with a mean age of 26 and 27 years respectively and an IQR of 24–30 for both periods.

The findings below capture providers’ perceptions of facilitators and barriers associated with the implementation of the INTERGROWTH-21^st^ standards as well as data on uptake and clinical decision-making. The results are divided into two main sections that include facility-level support for the introduction of the standards, and use and integration of the standards.

### Facility-level support for introduction of the standards

#### Training and buy-in

Providers reported that the standards helped foster independence for nurse-midwives by avoiding reliance on sonographers, allowing for birth preparedness, and increasing the quality of care provided. They also reported that collective problem-solving and open discussion of issues were instrumental and essential in facilitating their satisfaction and acceptance of the standards. Nurse-midwives noted that the main barriers to their buy-in were initial resistance to a new practice, lack of knowledge around the importance and utility of the standards and a short training period. They requested flexible training schedules and regular refresher trainings.

#### Job aids, reminders and resources

Job aids and training reminders put in place to facilitate the use of the INTERGROWTH-21^st^ standards included equipment (ultrasound machines, head circumference tapes and baby scales with measuring rods), new charting requirements, supportive software (estimated date of delivery (EDD) calculator application), paper-based aids (scan eligibility flowchart, equipment maintenance checklist, manuals and protocols), and in-person reminders (continuing medical education sessions, monthly team meetings and morning huddles). At both data collection time points during the implementation phase, the majority of providers reported that sufficient resources and job aids were available. Some providers expressed that additional equipment, promotional materials, flowcharts and instructions would be useful in enhancing the use and impact of the standards. At four months into the implementation phase, all three ultrasound providers asked that they receive compensation for the additional work. Jacaranda Health decided on a new service delivery model where one nurse-midwife conducted all gestational dating scans; her schedule was adjusted to allow her to focus only on the task at hand for which she was compensated.

“We feel that we are demoralized because despite the work you do you want to go a mile and do more scans but when you ask yourself you feel that I have not been paid for this and it’s making me tired and taking more of my time so I need some motivation.”

Ultrasound Nurse-Midwife (Implementation Phase– 4 months)

#### Knowledge and skills

Providers reported gaining knowledge and skills related to the administration and interpretation of ultrasounds, confirming and monitoring pregnancies, identifying and managing high-risk criteria and complications, and using newborn anthropometry equipment as a result of the training on and implementation of the INTERGROWTH-21^st^ standards. Moreover, providers noted that the routine use of the standards helped them to become more comfortable and confident in the care they offered. At the end of the study, all ten nurse-midwife interviewees who conducted newborn anthropometry reported being comfortable using the newborn tools.

“I would like to say that we are grateful for the skill [newborn anthropometry] because skill is something that is now in me and I feel empowered and so am grateful and we would like to have the practical skill for the ultrasound.”

Anthropometry Nurse-Midwife (Implementation Phase– 12 months)

This improvement in knowledge and skills was also reflected in the enhanced quality of ultrasound images over time. Analysis of variance and subsequent pairwise comparisons indicated that the quality scores improved significantly over time for head circumference and femur length. For both, we found that there was continuous and significant improvement from batch one to five which then continued to batch six.

### Use and integration of the standards

#### Workflow and workload

Over 86% of providers (25 of 29) surveyed at eight months into the implementation phase agreed or strongly agreed that the INTERGROWTH-21^st^ standards had become part of routine practice at Jacaranda Health. More than half of respondents (17 of 29) reported that the effort to integrate the tools into the clinical flow was worthwhile. The main reported facilitator to this integration was the change in the clinical workflow model to one dedicated nurse-midwife performing gestational dating ultrasounds. Reported barriers included long waiting times that turned away clients or resulted in complaints to already overwhelmed providers, especially in the first half of implementation; the time required to conduct procedures on top of regular clinical duties; and the lack of full-time availability of the nurse-midwife providing gestational dating services in the second half of the implementation phase.

At four months into the implementation phase, providers reported an initial increase in workload, but it was later normalized as they began to see the benefit of the standards and flow issues were addressed. At the end of the implementation phase, the majority of respondents (10 of 14) noted that the INTERGROWTH-21^st^ gestational dating and newborn size at birth standards had an overall positive effect on their workload. The expert sonographer at Jacaranda Health also expressed that the process of implementing the standards reduced her workload and allowed her to concentrate on fetal growth monitoring and other advanced obstetric scans. Similarly, other nurse-midwives reported feeling relieved that a dedicated provider was assigned to carry out all gestational dating ultrasounds; this adaptation eased the burden of combining gestational dating scans with their regular ANC duties, as originally planned and implemented.

“Initially, I can say it [the ultrasound scans] came as an extra tool without really knowing why I have to do this. But, through getting used to the tools and doing it regularly, I came to get used to it and think right now I can say it is something we feel like we cannot do without.”

Anthropometry Nurse-Midwife (Implementation Phase– 12 months)

Providers were already used to assessing newborns at birth, and reported that the standards increased their efficiency and delivery of accurate information.

#### Uptake of ultrasound standards

We observed a significant increase in the percentage of ANC clients receiving any obstetric ultrasounds (gestational dating or fetal growth monitoring), from 919 (53%) in the pre-implementation phase to 1156 (64%) in the implementation phase (p<0.0001). In total, 1217 obstetric ultrasound scans were performed in the pre-implementation phase and 1653 in the implementation phase. There was also a significant increase in the uptake of gestational dating ultrasounds in the implementation phase compared to the pre-implementation phase; from 373 scans representing 34% of eligible ANC clients to 580 scans representing 55% of eligible ANC clients (p<0.0001). The difference in uptake was further intensified when we changed from a multi-nurse model (40% of eligible ANC clients) to a dedicated ultrasound nurse model (73% of eligible ANC clients). Information on clients who chose to opt out of scans was only recorded during the implementation period so we were not able to compare opt-outs between the two study phases. With the implementation of the INTERGROWTH-21^st^ standards, documentation of the ultrasound form included six extra indicators related to the determination of the EDD via ultrasound and the LMP. Significantly more ultrasounds had complete documentation in the pre-implementation phase (96%) than the implementation phase (52%) (p<0.0001).

According to providers, the promotion of the gestational dating scan as free of charge during the implementation phase served as an incentive for eligible pregnant women to opt in and helped providers to advocate for the scan’s importance. Providers felt that the novelty and perception of the accuracy of the gestational dating services also facilitated their promotion and uptake.

“Because it has been free so there are no negative reactions but if it was coming with a cost then they [clients] would have said that am not prepared for it today and let me do it next time. Such things would crop up.”

Ultrasound Nurse-Midwife (Implementation Phase– 12 months)

At the same time, providers shared a number of issues that they felt needed to be tackled to further promote the uptake of the ultrasound standards. Reported demand side obstacles included long wait times; lack of print outs accompanying scan results to show family members; first ANC visits late in the pregnancy; and client misconceptions such as the fear that scans would harm the fetus. Client perceptions and experiences of care during the implementation of the standards are described in detail in another paper.

Perceived supply side barriers at four months into the implementation phase included lack of initial provider motivation to take on additional work; added requirements for documentation; long wait times that caused bottlenecks in providers’ schedules; longer time needed to conduct scans limiting the number of clients who could be seen; and an insufficient number of nurse-midwives trained in ultrasounds resulting in staff coverage gaps. At the end of implementation, providers noted that improvements were made as a result of the one-nurse model, but expressed concerns about the times when that designated nurse-midwife was off duty.

#### Use of newborn size at birth standards

All documented deliveries at Jacaranda Health, both live births and stillbirths, were enumerated for the study. Out of the 1092 newborns whose assessment forms indicated a newborn size at birth diagnosis in the implementation phase, 1037 (95%) were identified as being appropriate for gestational age (AGA), 25 (2%) as SGA and 30 (3%) as large for gestational age (LGA) using the new growth charts. When compared with the results generated by an online INTERGROWTH-21^st^ calculator utilized by data entry clerks, accuracy of diagnoses by nurse-midwives were found to be 76%, 80% and 77% for AGA, SGA and LGA respectively. The discrepancies were mainly due to the diagnosis falling between two diagnostic categories and incomplete documentation of the forms. Similar to the ultrasound form, there was a significantly lower (p < 0.0001) completeness of documentation of the newborn assessment form in the implementation phase (63%) compared to the pre-implementation phase (80%).

Generally, no major barriers were reported in the use of the newborn size at birth standards apart from some additional documentation needs. Reported facilitators for uptake of the newborn size at birth standards included enhanced infection control because the new scale was easy to clean and the head circumference tapes were disposable; decreased workload because weight and height could be measured on one apparatus; and increased accuracy and standardization. Finally, providers shared that the implementation of these standards built upon existing newborn anthropometry practices which facilitated their routine integration and helped to promptly identify newborn size at birth diagnoses.

#### Clinical decision-making

At eight months into the implementation phase, the majority of providers surveyed agreed that the integration of the INTERGROWTH-21^st^ standards improved overall growth monitoring (90%), high-risk identification and referral (76%) and quality of care (72%). While the proportion of ANC clients identified to have high-risk pregnancies and those referred (either internally to a Jacaranda Heath physician or externally to a tertiary care facility) increased slightly from the pre-implementation to the implementation phase, these increases were small and not statistically significant (Table 2). The information derived from the use of the ultrasound and newborn size at birth standards was reported to assist with the identification and management of high-risk pregnancies and births as well as timely referral to higher level care.

**Table 2:** Clinical decision-making outcomes.

Measure	Pre-implementation Phase N (%)	Implementation Phase N (%)	Comparison (p-value)
High-risk pregnancies	184 / 1744 (10.6%)	228 / 1807 (12.6%)	0.054
Inductions for post-date	82 / 1084 (7.6%)	34 / 1268 (2.7%)	<0.0001
Clients with ANC and delivery in one study phase	44 / 495 (8.9%)	20 / 587 (3.4%)	<0.0001
Clients with gestational dating ultrasound (and ANC) and delivery in one study phase	2 / 87 (2.3%)	3 / 113 (2.7%)	0.873
Deliveries by Cesarean section	245 / 1084 (22.6%)	300 / 1268 (23.7%)	0.544
Clients with ANC and delivery in one study phase	128 / 495 (25.9%)	123 / 587 (21.0%)	0.057
ANC clients referred (internally or externally)	190 / 1744 (10.9%)	224 / 1807 (12.40%)	0.163

Out of the 1084 deliveries in the pre-implementation phase, there were 82 (7.6%) inductions of labor due to post-date pregnancies (Table 2). This was significantly higher than those in the implementation phase, where only 34 (2.7%) of the 1268 deliveries had labor induced for this reason (p < 0.0001). A sub-analysis of Jacaranda Health clients who completed their pregnancy within one study phase yielded similar results (Table 2). However, a further sub-analysis for clients who received a gestational dating ultrasound during their first ANC visit in the pre-implementation phase compared to the implementation phase suggested that the gestational dating ultrasound itself may not have directly contributed to the reductions in induction rates for post-date. Clinic managers reported that a standard cut-off for post-date was instituted to facilitate the implementation of the standards; previously the cut-off for post-date was somewhat flexible and up to providers’ judgement. Despite the quantitative findings above, the nurse-midwife who performed all gestational dating scans in the second half of the implementation phase, felt that the ascertainment of EDDs using the standards was helpful in planning for timely delivery.

“[We are] getting almost accurate EDD’s to [help us] avoid unnecessary post-datism and thus it has helped us to act when necessary.”

Ultrasound Nurse-Midwife (Implementation Phase– 12 months)

When evaluating the association between the implementation of the standards and Cesarean section rates in one study phase versus the other, we did not find a statistically significant difference (Table 2). However, a few providers did report that performing obstetric scans allowed them to discourage clients from scheduling Cesarean sections on premature dates or dates based on family events rather than on an accurate understanding of gestational age. Additionally, during the implementation phase, clinic managers spent more time reminding their staff about the risks associated with overuse of medically non-indicated Cesarean sections.

The table below summarizes the main facilitators and barriers of implementing the standards as experienced by providers (Table 3).

**Table 3:** Facilitators and barriers of implementation perceived by providers.

Facilitators
Job aids and supportive protocols New knowledge and skills Perceived increase in the accuracy of EDDs Independence for nurse-midwives vs. reliance on sonographer Collective problem-solving and discussions Dedicated nurse-midwife performing gestational dating ultrasounds Prompt identification of high-risk clients and referrals Standardization of clinical practices Ultrasound scan provided at no cost Novelty of gestational dating services in geographical area New equipment
Barriers
Need to adapt clinical workflow Insufficient training and practice on ultrasound performance Lack of knowledge about importance/utility of standards Low motivation and no financial incentives for providers Staffing shortages Long queues for gestational dating ultrasound creating added pressure for provider Additional documentation requirements Late first ANC visits by clients

The majority of providers (13 of 16) expressed that the standards should continue to be used, highlighting how instrumental they have been in enhancing the quality of care as well as their potential to improve the health and survival of women and newborns.

## Discussion

The feasibility and acceptability of implementing the INTERGROWTH-21^st^ standards at Jacaranda Health were assessed through the examination of two broad dimensions: (1) facility-level support to introduce and integrate the standards into practice; and (2) use and integration of the standards to influence uptake of ultrasound and newborn size at birth standards, and clinical decision-making around pregnancy and delivery care. Facility-level support and provider buy-in proved to be critical factors driving the success of implementing the standards. Training and job aids were instrumental in the introduction and reinforcement of the standards. A literature review of clinical guideline implementation in a number of low resource settings also highlights the importance of introducing checklists and other job aids and making context-specific adjustments to foster integration [20]. In their paper introducing the rationale of the INTERGROWTH-21^st^ standards, Uauy and colleagues [21] stress the importance of training health providers and adapting clinical practices to facilitate successful implementation of the standards. Lack of or inadequate training on the provision of ultrasounds was discussed in a number of studies in low-and-middle income countries as one of the biggest challenges to the uptake of obstetric ultrasounds [9, 22–24]. Finding time for such training was a facility-level barrier experienced at Jacaranda Health as well as in other low resource settings; this was particularly difficult without disturbing clinical scheduling and interfering with the routine delivery of care while managing staff retention [22, 23].

Our implementation study sheds light on an innovative model of ultrasound delivery by nurse-midwives for whom the optimal training regimen has yet to be determined [23]. Nurse-led models of care, including the administration of ultrasounds, have been shown to be effective and warrant further exploration as WHO continues to encourage task-shifting in the provision of ANC [22, 25–28]. However, when task-shifting duties to nurses or midwives, an already overburdened cadre of health workers in low resource settings, health managers need to explore ways to do so without compromising job satisfaction, motivation, productivity, quality and safety. Ignatowicz and colleagues [29] similarly observed that building staff interest in a new initiative requires continuous engagement and alignment with changing provider goals and priorities. Learning about health provider perceptions has been found to be key in the successful introduction of new clinical processes [20].

We observed an increase in the uptake of obstetric ultrasounds, particularly gestational dating scans. However, only about 60% of ANC clients presented for their initial visit in the first two trimesters with most of these visits occurring in the second trimester. First ANC visits in the second and third trimesters are common in sub-Saharan Africa and lead to missed opportunities, during the first trimester, for the delivery of crucial components of ANC and management of obstetric complications [2, 30–32]. Although our study did not include a community behavior change component to increase demand for early ANC visits and awareness of the benefits of gestational dating, we believe that this would be helpful in addition to effective provider communication and identification of women eligible for gestational dating ultrasounds at the point of facility entry.

The use of the newborn size at birth standards was deemed feasible and acceptable by the nurse-midwives administering them; no significant barriers were reported. Unlike gestational dating ultrasounds, administration of these standards required minimal adaptations to existing newborn anthropometry practices while offering additional support for decision-making. Additionally, uptake was guaranteed to be universal since newborn anthropometry was a part of essential newborn care. Data on clinical decision-making indicators, when considered together with provider inputs on the standardization of post-date cut-offs and indications for Cesarean section during the study period, do not suggest a significant association between the implementation of the standards and clinical decision-making outcomes. Similar to our results, health workers in rural Kenya and Rwanda also reported that ultrasounds were instrumental in helping them to make clinical decisions, manage patients and improve the quality of ANC [22, 31]. Given that change in clinical indicators takes time and that other contextual factors were at play, namely the simultaneous adoption of the globally recognized SafeCare quality improvement standards to ensure quality and safety of patient care across various service areas [33], these findings are not surprising. It is possible that the routine implementation of the standards could further reduce rates of post-date induction and other clinical outcomes at Jacaranda Health and beyond. Whitworth and colleagues [6] and van Dyk and colleagues [34] found that pregnant women who received an early ultrasound were less likely to be induced for post-date. More time and research are needed to further explore the true clinical consequences of implementing the INTERGROWTH-21^st^ standards.

This is the first implementation study evaluating the feasibility and acceptability of the INTERGROWTH-21^st^ standards. Providers’ and clients’ experiences were equally prioritized and captured; this paper describes the former. Additional strengths include pre-implementation versus implementation comparisons, the use of both quantitative and qualitative methods and the setting of the study in a low resource context. This study also had a number of limitations. Our findings related to the ultrasound-based standards focus largely on gestational dating scans, a service that was not routinely provided before the study. Conversely, fetal growth monitoring was already part of routine care at Jacaranda Health; thus, we did not expect the implementation of the standards to influence a change but rather focused on overall uptake in obstetric ultrasounds. Results on the effect of the implementation of the standards on clinical decision-making outcomes were limited. We recommend that further time and financial investment would enable the standards to become engrained in clinical practice and lead to changes in maternal, perinatal and newborn outcomes, in particular at a low-risk health facility such as Jacaranda Health. The implementation process and its findings may not be generalizable to all health facilities in Kenya and beyond, but we have gleaned important insights that can facilitate adaptation and implementation of the full or partial INTERGROWTH-21^st^ package in other settings.

## Conclusions

This implementation study is timely as the INTERGROWTH-21^st^ standards are being adopted in a number of countries around the world and are proving to be an instrumental tool for the improvement of the quality of routine maternal and perinatal care and emergency situations that affect pregnancy and perinatal outcomes, such as the zika epidemic [14]. The standards integrate well with other aspects of ANC as recommended by the WHO and align with the WHO child growth standards [13]. Additionally, 75% of the currently available ultrasound machines now have INTERGROWTH-21^st^ standards built into them, thus making it more feasible to implement the standards on a larger scale. We hope that providers’ experiences and insights will be beneficial to facility managers who are considering, preparing for or in the process of implementing the INTERGROWTH-21^st^ standards or other similar clinical initiatives. This paper highlights the need to engage health providers in understanding and addressing their needs; the time and resource investment required for training and capacity building; the flexibility and monitoring necessary to facilitate adaptations to clinical flow; and the recognition that changes in clinical practice take time, especially when they deviate greatly from existing care. More research is needed to evaluate the financial, political and health implications of introducing routine gestational dating in low resource settings and community-based approaches to increase demand for early initiation of ANC.

## Supporting information

S1 File Qualitative guides (pre-implementation phase). (PDF)

S2 File Qualitative guides (implementation phase). (PDF)

S3 File Provider survey (implementation phase). (PDF)

S4 File Clinical data dorms. (PDF)
