# Supplementary material for: Implementing the INTERGROWTH-21st gestational dating and fetal and newborn growth standards in peri-urban Nairobi, Kenya: Provider experiences, uptake and clinical decision-making
Source: PLoS One. 2019 Mar 8;14(3):e0213388. doi: 10.1371/journal.pone.0213388 (PMC6407840; doi:10.1371/journal.pone.0213388)
Supplement: S1 File — (PDF) [file pone.0213388.s001.pdf]

S1 FILE. QUALITATIVE GUIDES: PRE-IMPLEMENTATION PHASE

S1.1 Pre-Implementation In-depth Interview: Clinic Manager

SECTION I: IMPLEMENTATION	
1.	How do you anticipate your staff will react to the introduction of these standards?<br><br>**Probe:** Will it be difficult to persuade the staff of the importance of adopting these standards? What can be introduced to reduce this reluctance?
2.	How do you think the introduction of the gestational dating scan will impact patient flow during antenatal care?<br><br>**Probe:** Do you think that the introduction of the scan will result in any bottlenecks in patient flow? If yes, please describe them. How will you address these bottlenecks?<br>If no, please describe why not. What measures have been taken to prevent possible bottlenecks.
3.	How many scans do patients currently expect to receive during their antenatal care?<br><br>**Probe:** Please describe your opinion on patient attitudes towards ultrasound scans.<br>**Probe:** Will patients be reluctant to receive multiple scans?<br>If yes, what do you think will be the major factors influencing the resistance? What steps can be taken to overcome this reluctance?
4.	Having received a gestational dating scan, how likely do you think patients will return for an anatomy scan?
5.	Can you think of any other major issues that providers at Jacaranda Health are likely to face while implementing the standards?

SECTION II: SATISFACTION	
6.	How do you think the implementation of these standards will impact clinical decision making?<br><br>**Probe:** If you think these standards will not improve decision making, please describe why.
7.	Do you think that the implementation of these standards will have any impact on the accuracy of gestational dating in the clinic? Please explain.<br><br>**Probe:** If you not, please describe why not and outline any other appropriate solutions.
8.	Do you think that the implementation of these standards will have any impact on the way women are referred for high risk pregnancy management? Please explain.<br><br>**Probe:** If not, please describe why not and outline any other appropriate solutions.
9.	Do you think that the implementation of these standards will have any impact on better management of complications during pregnancy? Please explain.<br><br>**Probe:** If not, please describe why and outline any other appropriate solutions.
10.	Do you think that the implementation of INTERGROWTH-21st standards will positively or negatively impact patient satisfaction? Please explain.<br><br>**Probe:** How do you think patients will feel about longer waiting times?<br>**Probe:** How do you think patients will feel about getting a free ultrasound?

SECTION III: NEWBORN ASSESSMENT	
11.	What do you think are the major barriers to assessing gestational age and appropriate newborn weight?
12.	What maternal and fetal conditions during pregnancy and at birth make you worry about an infant's risk for illness?
13.	Do you think that the implementation of these standards will have any impact on the accuracy of assessment of newborn size at birth? Please explain.<br><br>**Probe:** If you think these standards will not improve accuracy, please describe why not and outline any other appropriate solutions.

S1.2 Pre-Implementation In-depth Interview: Nurse-Midwife (Newborn Anthropometry)

SECTION I: GENERAL QUESTIONS	
1.	In your opinion, what are the most important aspects of antenatal care for pregnant women?
2.	How prepared do you think you are in offering different antenatal services?
3.	Are the protocols for antenatal care defined clearly and used systematically?
4.	Please describe the barriers, if any, to the provision of antenatal care for clients at Jacaranda Health?
5.	Please describe the barriers, if any, to determining if a woman should be categorized as high risk and referred at any point during a pregnancy?
6.	To what extent are you able to manage various medical complications associated with pregnancy?
7.	Describe the factors that affect patient satisfaction during antenatal visits and deliveries?

SECTION II: GESTATIONAL DATING	
8.	Please describe the current method used for gestational dating at Jacaranda Health.
9.	How comfortable do you feel with the current method used for gestational dating at Jacaranda Health?
10.	Do you face any difficulties with the current method used for gestational dating at Jacaranda Health?<br><br>**Probe:** Do you feel like you have all the tools you require for dating a fetus accurately?<br>**Probe:** If not, what kind of tools do you think would help you date a fetus accurately?
11.	Are you able to accurately predict the estimated delivery date (EDD) of the women who attend antenatal care?
12.	How does accurate gestational dating impact your clinical decision-making process and case management?<br><br>**Probe:** What is the impact of gestational dating on your decision-making around preterm births, induction of labor and decisions to conduct Cesarean sections?

SECTION III: NEWBORN CARE	
13.	How does the current gestational dating methodology affect your ability to determine if a baby is the appropriate size for his/her gestational age?<br><br>**Probe:** Are you able to determine if the baby is large for gestational age, appropriate for gestational age or small for gestational age? Can you describe the process by which you make this determination?
14.	How do you determine if an infant is an appropriate weight?
15.	What are barriers to assessing gestational age and appropriate weight for newborns?
16.	How does the current gestational dating methodology affect your ability to determine if a baby is preterm?

17.	Please describe how you would assess the level of care required for a preterm baby?<br><br>**Probe:** Do you feel comfortable with assessing the level of care required for a preterm baby?<br>**Probe:** If not, please describe the barriers you face for assessing the required level of care.

**SECTION IV: EXPERIENCE WITH ULTRASOUNDS**	
18.	Please describe any training you have received in performing ultrasounds on pregnant women?<br><br>**Probe:** Have you ever performed ultrasounds on pregnant women? If so, for how long? How long ago?
19.	Please describe how the obstetric ultrasound is used in the clinical management of a pregnancy?

S1.3 Pre-Implementation Focus Group Discussion: Nurse-Midwife (Newborn Anthropometry)

Moderator: Thank you for participating in this focus group today. My name is _________ and I will be moderating this conversation. We are here to talk about your thoughts about service provision at Jacaranda Health. We would like to focus on your experiences in the provision of antenatal care and delivery care to clients. __________ will be taking notes and recording the conversation, so we can remember your comments accurately after the discussion is over. Only the research team will have access to these notes and recordings and we will remove identifiable information. The research team will not share any information associated with you with other Jacaranda staff.

Does anyone have any questions before we begin?

In order to have a more positive conversation today I'd like to set a few ground rules:

- Please respect the other group members by not discussing details of the content of the discussion once you leave the focus group site. I want to remind you of the informed consent you signed and that you have agreed not to disclose anything concerning their participation in the study with anyone other than the research team.
- Please speak one person at a time. Each person's thoughts and opinions are very valuable. Speaking one at a time lets everyone hear and react and it makes the note-taker's job easier.
- Please also respect each person's opinions even if they disagree with your own. This means not doing anything that could cause another member of the group to feel uncomfortable. All participants need to feel free to express their opinions without criticism or judgment.

SECTION I: ANTENATAL AND DELIVERY CARE

Moderator: To begin this conversation, I would like to ask you some general questions about your experiences during the provision of antenatal and delivery care at Jacaranda Health.

- Please talk about your experiences providing antenatal care to pregnant women at Jacaranda Health.
    - **Probe:** Do you face any problems in care provision? If yes, please describe.
    - **Probe:** How is patient flow?
    - **Probe:** What are the most important aspects of quality of care?
    - **Probe:** Are you satisfied with the level of service you are able to provide to patients?

- Please describe your experiences with women categorized as high-risk during antenatal care?
    - **Probe:** Do you feel prepared to refer a woman to a high-risk provider?
    - **Probe:** What training have you received in high-risk referral?
    - **Probe:** If you do not feel prepared to refer a woman, please describe the barriers to determining if a woman should be categorized as high risk.

SECTION II: GESTATIONAL DATING

Moderator: Now, I would like to speak with you about your experience with determining the estimated delivery date (EDD) of a woman who attends an antenatal care visit.

- Please describe the current method you use for gestational dating of a pregnancy at Jacaranda Health.
    - **Probe:** How comfortable do you currently feel with the method you use for gestational dating?
    - **Probe:** Do you feel that this method is accurate?

- How does the determination of EDD affect your clinical decision-making process and case management?

SECTION III: NEWBORN CARE

Moderator: Now I would like to speak with you about your experiences with newborn care.

- Does the newborn's size at birth have any impact on your clinical decision-making regarding the level of care required for that newborn?
    - **Probe:** What do the terms small for gestational age, appropriate for gestational age and large for gestational age mean to you?
    - **Probe:** Are you able to determine if a baby is small for gestational age, appropriate for gestational age and large for gestational age? If yes, can you describe the process by which you make this determination?

- Please describe how you would assess the level of care required for a preterm baby?
    - **Probe:** Do you feel comfortable with assessing the level of care required for a preterm baby? If not, please describe the barriers you face.

S1.4 Pre-Implementation In-depth Interview: Nurse-Midwife (Ultrasound)

SECTION I: GENERAL QUESTIONS	
1.	In your opinion, what are the most important aspects of antenatal care for pregnant women?
2.	How prepared do you think you are in offering different antenatal services?
3.	Are the protocols for antenatal care defined clearly and used systematically?
4.	Please describe the barriers, if any, to the provision of antenatal care for clients at Jacaranda Health?
5.	Please describe the barriers, if any, to determining if a woman should be categorized as high risk and referred at any point during a pregnancy?
6.	To what extent are you able to manage various medical complications associated with pregnancy?
7.	Describe the factors that affect patient satisfaction during antenatal visits and deliveries?

SECTION II: GESTATIONAL DATING	
8.	Please describe the current method used for gestational dating at Jacaranda Health.
9.	How comfortable do you feel with the current method used for gestational dating at Jacaranda Health?
10.	Do you face any difficulties with the current method used for gestational dating at Jacaranda Health?<br><br>**Probe:** Do you feel like you have all the tools you require for dating a fetus accurately?<br>**Probe:** If not, what kind of tools do you think would help you date a fetus accurately?
11.	Are you able to accurately predict the estimated delivery date (EDD) of the women who attend antenatal care?
12.	How does accurate gestational dating impact your clinical decision-making process and case management?<br><br>**Probe:** What is the impact of gestational dating on your decision-making around preterm births, induction of labor and decisions to conduct Cesarean sections?

SECTION III: FETAL GROWTH MONTIORING	
13.	Please describe the current method used for fetal growth monitoring at Jacaranda Health.
14.	How comfortable do you feel with the current method used for fetal growth monitoring at Jacaranda Health?
15.	Do you face any difficulties with the current method used for fetal growth monitoring at Jacaranda Health?<br><br>**Probe:** Do you feel like you have all the tools you require to monitor fetal growth?<br>**Probe:** If not, what kind of tools do you think would help you to monitor fetal growth?
16.	How does accurate fetal growth monitoring impact your clinical decision-making process and case management?

	**Probe:** What is the impact of fetal growth monitoring on your decision-making around preterm births, induction of labor and decisions to conduct Cesarean sections?

**SECTION IV: NEWBORN CARE**	
17.	How does the current gestational dating methodology affect your ability to determine if a baby is the appropriate size for his/her gestational age?<br><br>**Probe:** Are you able to determine if the baby is large for gestational age, appropriate for gestational age or small for gestational age? Can you describe the process by which you make this determination?
18.	How do you assess an infant's gestational age at birth?
19.	How do you determine if an infant is an appropriate weight?
20.	What are barriers to assessing gestational age and appropriate weight for newborns?
21.	How does the current gestational dating methodology affect your ability to determine if a baby is preterm?
22.	Please describe how you would assess the level of care required for a preterm baby?<br><br>**Probe:** Do you feel comfortable with assessing the level of care required for a preterm baby?<br>**Probe:** If not, please describe the barriers you face for assessing the required level of care.

S1.5 Pre-Implementation In-depth interview: Physician

SECTION I: ANTENATAL CARE PROVISION	
1.	Please describe your opinion of the quality of care provided by the antenatal care providers at Jacaranda Health.
2.	Do you think the antenatal care providers are able to accurately predict the estimated delivery date (EDD) of the women who attend antenatal care?<br><br>**Probe:** Please describe the barriers to accurate prediction of EDD, if any.
3.	Does the current gestational dating methodology have any impact (positive or negative) on case management during antenatal care? Please explain.

SECTION II: HIGH-RISK CARE PROVISION	
4.	Please describe the process of high-risk referral at Jacaranda Health.
5.	Currently, do you think that all women at high-risk for complications during pregnancy are referred accurately by the antenatal care providers?<br><br>**Probe:** What are the barriers to accurate high-risk referral at Jacaranda Health?<br>**Probe:** Please describe possible solutions to the barriers to accurate high-risk referral.
6.	Please describe the current method used at Jacaranda Health for fetal growth monitoring.<br><br>**Probe:** What are the barriers to accurate fetal growth monitoring at Jacaranda Health?<br>**Probe:** Please describe possible solutions to facilitate accurate fetal growth monitoring at Jacaranda Health.
7.	How does accurate fetal growth monitoring impact your clinical decision-making process and case management?<br><br>**Probe:** What is the impact of fetal growth monitoring on your decision-making around preterm births, induction of labor and decisions to conduct Cesarean sections?

SECTION III: POTENTIAL IMPACT OF IMPLEMENTATION	
8.	How do you think the implementation of the INTERGROWTH 21st standards will impact the accuracy of gestational dating?
9.	How do you think the implementation of the INTERGROWTH 21st standards will impact the accuracy of fetal growth monitoring?
10.	How do you think the implementation of the INTERGROWTH 21st standards will impact clinical case management of high-risk patients at Jacaranda Health?
