# Supplementary material for: Implementing the INTERGROWTH-21st gestational dating and fetal and newborn growth standards in peri-urban Nairobi, Kenya: Provider experiences, uptake and clinical decision-making
Source: PLoS One. 2019 Mar 8;14(3):e0213388. doi: 10.1371/journal.pone.0213388 (PMC6407840; doi:10.1371/journal.pone.0213388)
Supplement: S2 File — (PDF) [file pone.0213388.s002.pdf]

S2 FILE. QUALITATIVE GUIDES: IMPLEMENTATION PHASE (4 & 12 MONTHS)

S2.1 Implementation In-depth Interview: Clinic Manager

READ OUT AT 4 MONTHS: Over the past 4 months, the INTERGROWTH-21st project has been implemented at Jacaranda Health. I would like to speak to you about your experience and attitudes towards the project over the last 4 months.

READ OUT AT 12 MONTHS: Over the past year, the INTERGROWTH-21st project has been implemented at Jacaranda Health. I would like to speak to you about your experience and attitudes towards the project over the last 8 months since we last spoke.

**SECTION I: TRAINING – APPROPRIATENESS & PENETRATION**	
1.	**ASK AT 4 MONTHS ONLY:**<br>Please describe the key components of the training on INTERGROWTH-21st tools.
2.	**ASK AT 4 MONTHS ONLY:**<br>Please share your overall impressions of the trainings.<br><br>**Probe:** What did you find useful about the trainings? Explain why.<br>**Probe:** What would you change/improve about the training?
3.	**ASK AT 4 MONTHS ONLY:**<br>How did the training on INTERGROWTH-21st tools affect the regular workflow of the clinic?<br><br>**Probe:** Were there any issues with the scheduling of staff for training?<br>**Probe:** How did you address these scheduling issues?<br>**Probe:** Were there any negative impacts of the training on regular workflow?
4.	**ASK AT 4 MONTHS ONLY:**<br>Do you feel that the INTERGROWTH-21st trainings prepared staff to use the tools?<br><br>**Probe:** Did staff understand the rationale for the implementation of the standards?<br>**Probe:** Do you feel like the trainings fully prepared staff to perform and interpret ultrasounds?<br>If yes, please describe how this was facilitated.<br>If no, please describe why.<br>**Probe:** Do you feel like the trainings fully prepared staff to perform and interpret newborn size at birth measurements and diagnoses using the new tools?<br>If yes, please describe how this was facilitated.<br>If no, please describe why.
5.	What procedures/aids have been put in place to remind staff of the training and use of INTERGROWTH-21st tools?<br><br>**Probe:** Are they being used by staff? Please comment on the procedures/aids that are most and least useful.<br>**Probe:** Are they sufficient to remind staff of clinical procedures?<br>**Probe:** Are there areas where job aids/reminders are not present and would be helpful?<br>**Probe (12 MONTHS ONLY):** Were there any refresher trainings? If yes, please describe.

SECTION II: IMPLEMENTATION – ACCEPTABILITY & FEASIBILITY	
6.	How did your staff feel about the introduction of these tools into antenatal and postnatal care?<br><br>**Probe:** Did you encounter any resistance in the introduction of the tools?<br>**Probe:** Was it difficult to persuade the staff of the importance of adopting these standards? If yes, how did you address this?<br>**Probe:** Are there any measures that could have aided in smoother introduction of these tools?
7.	Please describe the process by which the gestational dating scan was integrated into the workflow of a regular antenatal care visit.<br><br>**Probe:** Please describe what facilitated integration.<br>**Probe:** Please describe any barriers to integration. How were they addressed?
8.	How do you think that the implementation of the INTERGROWTH-21st tools has impacted patient satisfaction?<br><br>**Probe:** What have been the facilitators?<br>**Probe:** What have been the barriers?<br>**Probe:** How did patients react to new clinical recommendations around ultrasound scans? Was there resistance? If yes, why and how was it addressed?
9.	How has the implementation of the INTERGROWTH-21st tools impacted your daily workload? Please explain.
10.	Can you please describe any other major issues/barriers that you or your staff faced while implementing the tools?<br><br>**Probe:** Please describe the measures you adopted to solve them.

SECTION III: IMPLEMENTATION - APPROPRIATENESS	
11.	How have the INTERGROWTH-21st tools impacted the accuracy of gestational dating, fetal growth estimation and newborn size assessment in the clinic?<br><br>**Probe:** What about the tools had a positive impact on accuracy of these measurements?<br>**Probe:** What about the tools had a negative impact on accuracy of these measurements?
12.	How has the implementation of the INTERGROWTH-21st tools impacted clinical decision-making related to the identification and management of high-risk pregnancies and newborns?<br><br>**Probe:** Did the tools help to better identify high-risk women?<br>**Probe:** Did the tools help to facilitate more appropriate referrals of high-risk pregnancies?<br>**Probe:** Did the tools help to better identify high-risk newborns?<br>**Probe:** Did the tools help to facilitate more appropriate referrals of high-risk newborns?
13.	Do you think that staff have all the resources they need for appropriate utilization of the INTERGROWTH 21st tools?<br><br>**Probe:** If not, what would help you?

14.	**ASK AT 12 MONTHS ONLY:**<br>What do you envision as the future of INTERGROWTH-21st at Jacaranda Health?<br><br>**Probe:** Do you think Jacaranda Health should continue to utilize the INTERGROWTH-21st tools? Why or why not?
15.	**ASK AT 12 MONTHS ONLY:**<br>If Jacaranda Health continues to use the INTERGROWTH-21st tools, do you think the gestational dating scans should remain free?<br><br>**Probe:** Why or why not?

THANK YOU FOR TAKING THE TIME TO SPEAK WITH ME TODAY AND SHARING YOUR EXPERIENCE. IS THERE ANYTHING ELSE YOU WOULD LIKE TO SHARE WITH ME ABOUT YOUR EXPERIENCE?

S2.2 Implementation In-depth Interview: Nurse Midwife (Newborn Anthropometry)

READ OUT AT 4 MONTHS: Over the past 4 months, the INTERGROWTH-21st project has been implemented at Jacaranda Health. I would like to speak to you about your experience and attitudes towards the project over the last 4 months.

READ OUT AT 12 MONTHS: Over the past year, the INTERGROWTH-21st project has been implemented at Jacaranda Health. I would like to speak to you about your experience and attitudes towards the project over the last 8 months since we last spoke.

**SECTION I: TRAINING**	
1.	**ASK AT 4 MONTHS ONLY:**<br>Please describe the key components of the training on INTERGROWTH-21st tools.
2.	**ASK AT 4 MONTHS ONLY:**<br>What were your overall impressions of the trainings that you attended?<br><br>**Probe:** What did you find useful about the trainings? Please explain why.<br>**Probe:** Were there any gaps in the training?<br>**Probe:** Was the amount of time spent on the trainings adequate? If not, how much more time do you think would be needed?<br>**Probe:** What would you change/improve about the training?
3.	**ASK AT 4 MONTHS ONLY:**<br>What did the trainings teach you about the rationale for the implementation of the INTERGROWTH-21st tools?
4.	**ASK AT 4 MONTHS ONLY:**<br>After the trainings, how prepared did you feel to refer women for high-risk care in your everyday practice?<br><br>**Probe:** If you felt prepared, please explain what parts of the training were particularly helpful to make you feel that way.<br>**Probe:** If you did not feel prepared, please explain what parts of the training you did not feel prepared to implement and why.
5.	**ASK AT 4 MONTHS ONLY:**<br>After the trainings, how prepared did you feel to assess newborn size at birth and newborn health in your everyday practice?<br><br>**Probe:** If you felt prepared, please explain what parts of the training were particularly helpful to make you feel that way.<br>**Probe:** If you did not feel prepared, please explain what parts of the training you did not feel prepared to implement and why.
6.	What procedures/aids have been put in place to remind staff of the training and use of INTERGROWTH-21st tools?<br><br>**Probe:** Are you using them?<br>**Probe:** Which procedures/aids are most useful?<br>**Probe:** Are they sufficient to remind you of the INTERGROWTH procedures?<br>**Probe:** Are there areas where job aids/reminders are not present and would help you?<br>**Probe (12 MONTHS ONLY):** Were there any refresher trainings? If yes, please describe.

SECTION II: IMPLEMENTATION OF PROTOCOLS	
7.	Please describe the process by which the high-risk referral protocols were integrated into regular case management?<br><br>**Probe:** Please describe what facilitated integration.<br>**Probe:** Please describe any barriers to integration. How were they addressed?
8.	Please describe the process by which the newborn size at birth protocols and tools were integrated into regular case management of newborns?<br><br>**Probe:** Please describe what facilitated integration.<br>**Probe:** Please describe any barriers to integration. How were they addressed?
9.	Please describe the process of the integration of the gestational dating scans in the regular antenatal care visit workflow?<br><br>**Probe:** Please describe what facilitated integration.<br>**Probe:** Please describe any barriers to integration. How were they addressed?
10.	What impact did the integration of the gestational dating scans into the antenatal care visit have on patient waiting times?
11.	How do you think that the implementation of the INTERGROWTH-21st tools has impacted patient satisfaction? Please explain.<br><br>**Probe:** Specifically, for newborn size at birth measurement, how have women reacted to the diagnoses of their babies if they were small or large for gestational age?
12.	How has the implementation of the INTERGROWTH-21st tools impacted your daily workload? Please explain.
13.	How has the use of INTERGROWTH-21st tools influenced your clinical skills and knowledge? Please explain.
14.	How comfortable do you feel using the INTERGROWTH-21st tools in your clinical practice? Please explain.
15.	How has the integration of gestational dating scans affected case management and clinical decision-making related to the identification of high-risk pregnant women and their appropriate referral?<br><br>**Probe:** How did the new way of determining the estimated delivery date (EDD) of a woman through gestational dating ultrasound affect your ability to predict EDD, manage pregnancy complications and make clinical decisions?<br>**Probe:** Did the tools help better identify high-risk women?<br>**Probe:** Did the tools help facilitate more appropriate referrals?
16.	How has the integration of newborn size at birth INTERGROWTH-21st tools affected case management and clinical decision-making related to the accurate measurement of newborn size, identification of high-risk newborns and their appropriate referral?<br><br>**Probe:** How did the new way of assessing newborn size and diagnosis affect your ability to manage newborns at risk for complications and make clinical decisions?

	**Probe:** Did the tools help better identify high-risk newborns?<br>**Probe:** Did the tools help facilitate more appropriate referrals of high-risk newborns?
17.	Do you think that you have all the resources you need for appropriate utilization of the INTERGROWTH-21st tools?<br><br>**Probe:** If not, what would help you?
18.	**ASK AT 12 MONTHS ONLY:**<br>Do you think Jacaranda should continue to utilize the Intergrowth tools? Why or why not?<br><br>**Probe:** Do you think the gestational scan should continue to be free or should clients begin to pay for the service?

THANK YOU FOR TAKING THE TIME TO SPEAK WITH ME TODAY AND SHARING YOUR EXPERIENCE. IS THERE ANYTHING ELSE YOU WOULD LIKE TO SHARE WITH ME ABOUT YOUR EXPERIENCE?

S2.3 Implementation Focus Group Discussion: Nurse-Midwife (Newborn Anthropometry)

READ OUT AT 4 MONTHS ONLY: Thank you for participating in this focus group today. My name is _________ and I will be moderating this conversation. We are here to assess your attitudes about and experiences with implementing the INTERGROWTH-21st tools at Jacaranda over the past 4 months. This has included gestational dating by ultrasound, fetal growth assessment by ultrasound and newborn size at birth. Since you have been the primary users of these tools, we would value your feedback and advice. __________ will be taking notes and recording the conversation so we can remember your comments accurately after the discussion is over. Only the research team will have access to these notes and recording and we will remove identifiable information. The research team will not share any information associated with you with other Jacaranda staff.

READ OUT AT 12 MONTHS ONLY: Thank you for participating in this focus group today. My name is _________ and I will be moderating this conversation. We are here to assess your attitudes about and experiences with implementing the INTERGROWTH-21st tools at Jacaranda over the past year. This includes gestational dating by ultrasound, fetal growth assessment by ultrasound and newborn size at birth. Since you have been the primary users of these standards, we would value your feedback and advice. Most if not all of you will recall that we spoke about 8 months ago; I would like to hear about your thoughts now and over the past 8 months. __________ will be taking notes and recording the conversation, so we can remember your comments accurately after the discussion is over. Only the research team will have access to these notes and recording and we will remove identifiable information. The research team will not share any information associated with you with other Jacaranda staff.

READ OUT AT 4 & 12 MONTHS:

Does anyone have any questions before we begin?

In order to have a more positive conversation today I'd like to set a few ground rules:

- Please respect the other group members by not discussing details of the content of the discussion once you leave the focus group site. I want to remind you of the informed consent you signed and that you have agreed not to disclose anything concerning others' participation in the study with anyone other than the research team.
- Please speak one person at a time. Each person's thoughts and opinions are very valuable. Speaking one at a time lets everyone hear and react and it makes the note-taker's job easier.
- Please also respect each person's opinions even if they disagree with your own. This means not doing anything that could cause another member of the group to feel uncomfortable. All participants need to feel free to express their opinions without criticism or judgment.

SECTION I: TRAINING

Moderator: To begin, I would like to speak with you about your experiences during the trainings that you received before the implementation of the tools. Please talk about each set of trainings (high risk, anthropometry, accurate dating of pregnancy) separately.

1. **ASK AT 4 MONTHS ONLY:** Please describe the key components of the training on the INTERGROWTH-21st tools.

2. **ASK AT 4 MONTHS ONLY:** What were your overall impressions of the trainings that you attended?

   Probe: What did you find useful about the trainings? Please explain why.
   Probe: Were there any gaps in the training?
   Probe: Was the amount of time spent on the trainings adequate? If not, how much more time do you think would be needed?
   Probe: What would you change/improve about the training?

3. **ASK AT 4 MONTHS ONLY:** What did the trainings teach you about the rationale for the implementation of the INTERGROWTH-21st tools?

4. **<u>ASK AT 4 MONTHS ONLY:</u>** After the trainings, how prepared did you feel to refer women for high-risk care in your everyday practice?

    Probe: If you felt prepared, please explain what parts of the training were particularly helpful to make you feel that way.
    Probe: If you did not feel prepared, please explain what parts of the training you did not feel prepared to implement and why.

5. **<u>ASK AT 4 MONTHS ONLY:</u>** After the trainings, how prepared did you feel to assess newborn size at birth and newborn health in your everyday practice?

    Probe: If you felt prepared, please explain what parts of the training were particularly helpful to make you feel that way.
    Probe: If you did not feel prepared, please explain what parts of the training you did not feel prepared to implement and why.

6. What procedures/aids have been put in place to remind you of the training and use of the INTERGROWTH-21st tools?

    Probe: Are you using them?
    Probe: Which procedures/aids are most useful?
    Probe: Are they sufficient to remind you of the INTERGROWTH procedures?
    Probe: Are there areas where job aids/reminders are not present and would help you?
    Probe (12 MONTHS ONLY): Were there any refresher trainings? If yes, please describe.

SECTION II: IMPLEMENTATION OF PROTOCOLS

Moderator: I would like to now talk about your experience of implementing the tools.

7. Please describe the process by which the high-risk referral protocols were integrated into regular case management?

    Probe: Please describe what facilitated integration.
    Probe: Please describe any barriers to integration. How were they addressed?

8. Please describe the process by which the newborn size at birth protocols and tools were integrated into regular case management of newborns?

    Probe: Please describe what facilitated integration.
    Probe: Please describe any barriers to integration. How were they addressed?

9. Please describe the process of the integration of the gestational dating scans in the regular antenatal care visit workflow?

   Probe: Please describe what facilitated integration.
   Probe: Please describe any barriers to integration. How were they addressed?

10. What impact did the integration of the gestational dating scans into the antenatal care visit have on patient waiting times?

11. How do you think that the implementation of the INTERGROWTH-21st tools has impacted patient satisfaction? Please explain.
    Probe: Specifically, for newborn size at birth measurement, how have women reacted to the diagnoses of their babies if they were small or large for gestational age?

12. How has the implementation of the INTERGROWTH-21st tools impacted your daily workload? Please explain.

13. How has the use of INTERGROWTH-21st tools influenced your clinical skills and knowledge? Please explain.

14. How comfortable do you feel using the INTERGROWTH-21st tools in your clinical practice? Please explain.

15. How has the integration of gestational dating scans affected case management and clinical decision-making related to the identification of high-risk pregnant women and their appropriate referral?

    Probe: How did the new way of determining the estimated delivery date (EDD) of a woman through gestational dating ultrasound affect your ability to predict EDD, manage pregnancy complications and make clinical decisions?
    Probe: Did the tools help better identify high-risk women?
    Probe: Did the tools help facilitate more appropriate referrals?

16. How has the integration of newborn size at birth INTERGROWTH-21st tools affected case management and clinical decision-making related to the accurate measurement of newborn size, identification of high-risk newborns and their appropriate referral?

    Probe: How did the new way of assessing newborn size and diagnosis affect your ability to manage newborns at risk for complications and make clinical decisions?
    Probe: Did the tools help better identify high-risk newborns?
    Probe: Did the tools help facilitate more appropriate referrals of high-risk newborns?

17. Do you think that you have all the resources you need for appropriate utilization of the INTERGROWTH-21st tools?

    Probe: If not, what would help you?

18. **ASK AT 12 MONTHS ONLY:** Do you think Jacaranda should continue to utilize the Intergrowth tools? Why or why not?

    Probe: Do you think the gestational scan should continue to be free or should clients begin to pay for the service?

THANK YOU FOR TAKING THE TIME TO SPEAK WITH ME TODAY AND SHARING YOUR EXPERIENCE. IS THERE ANYTHING ELSE YOU WOULD LIKE TO SHARE WITH ME ABOUT YOUR EXPERIENCE?

S2.4 Implementation In-depth Interview: Nurse-Midwife (Ultrasound)

READ OUT AT 4 MONTHS: Over the past 4 months, the INTERGROWTH-21st project has been implemented at Jacaranda Health. I would like to speak to you about your experience and attitudes towards the project over the last 4 months including training that was conducted prior to implementation.

READ OUT AT 12 MONTHS: Over the past year, the INTERGROWTH-21st project has been implemented at Jacaranda Health. You may recall that we spoke about your experience 4 months after the start of implementation. I would now like to speak to you about your experience and attitudes towards the project over the last 8 months since we last spoke.

**SECTION I: TRAINING – APPROPRIATENESS & PENETRATION**	
1.	<u>**ASK AT 4 MONTHS ONLY:**</u><br>Please describe the key components of the training on INTERGROWTH-21st tools.
2.	<u>**ASK AT 4 MONTHS ONLY:**</u><br>Please share your overall impressions of the trainings.<br><br>**Probe:** What did you find useful about the trainings? Explain why.<br>**Probe:** What would you change/improve about the training?
3.	<u>**ASK AT 4 MONTHS ONLY:**</u><br>What did the trainings teach you about the rationale for the implementation of the INTERGROWTH-21st tools?
4.	<u>**ASK AT 4 MONTHS ONLY:**</u><br>After the trainings, how prepared did you feel to implement what you had learned in your everyday practice?<br><br>**Probe:** If you felt prepared, please explain what parts of the training were particularly helpful to make you feel that way.<br>**Probe:** If you did not feel prepared, please explain what aspects of care you did not feel prepared to implement and why.
5.	What procedures/aids have been put in place to remind staff of the training and use of INTERGROWTH-21st tools?<br><br>**Probe:** Are you using them?<br>**Probe:** Which procedures/aids are most useful?<br>**Probe:** Are they sufficient to remind you of the INTERGROWTH-21st procedures?<br>**Probe:** Are there areas where job aids/reminders are not present and would help you?<br>**Probe (12 MONTHS ONLY):** Have you attended any refresher trainings? If yes, please explain.

**SECTION II: IMPLEMENTATION – ACCEPTABILITY & FEASIBILITY**	
6.	Please describe the process by which the gestational dating scan was integrated into the workflow of a regular ANC visit.<br><br>**Probe:** Please describe what facilitated integration.<br>**Probe:** Please describe any barriers to integration. How were they addressed?
7.	How has the implementation of the INTERGROWTH-21st tools impacted your daily workload? Please explain.

8.	What impact did the integration of the gestational dating scans into the antenatal care visit have on patient waiting times?
9.	How have patients reacted to the new clinical recommendations around ultrasound scans?<br><br>**Probe:** Did you encounter resistance? If yes, please describe the reasons and how they were overcome.<br>**Probe:** Please highlight any positive feedback.
10.	How do you think that the implementation of the INTERGROWTH-21st tools has impacted patient satisfaction? Please explain.
11.	How has the use of INTERGROWTH-21st tools influenced your clinical skills and knowledge? Please explain.
12.	How comfortable do you feel using the INTERGROWTH-21st tools in your clinical practice? Please explain.

**SECTION III: IMPLEMENTATION - APPROPRIATENESS**	
13.	Do you explain the purpose of the tools to the patients who are eligible for the gestational dating scan?<br><br>**Probe:** If no, why not?<br>**Probe:** If yes, how do you explain it?<br>**Probe:** If yes, do you feel that patients understand and accept the explanation?<br>**Probe:** If yes, have patients declined an ultrasound after your explanation? If so, why?
14.	Have the INTERGROWTH-21st tools impacted the accuracy of gestational dating and fetal growth estimation? Please explain.<br><br>**Probe:** If yes, how?<br>**Probe:** If no, why not?
15.	Have the INTERGROWTH-21st tools impacted the accuracy of newborn size assessment?<br><br>**Probe:** If yes, how?<br>**Probe:** If no, why not?
16.	How has the implementation of the INTERGROWTH-21st tools impacted clinical decision-making related to the identification and management of high-risk pregnancies and newborns?<br><br>**Probe:** Did the tools help to better identify high-risk women?<br>**Probe:** Did the tools help to facilitate more appropriate referrals of high-risk pregnancies?<br>**Probe:** Did the tools help to better identify high-risk newborns?<br>**Probe:** Did the tools help to facilitate more appropriate referrals of high-risk newborns?
17.	Do you think that staff have all the resources they need for appropriate utilization of the INTERGROWTH 21st tools?<br><br>**Probe:** If not, what would help you?
18.	**<u>ASK AT 12 MONTHS ONLY:</u>**<br>What do you envision as the future of the INTERGROWTH-21st ultrasound tools at Jacaranda Health?<br><br>**Probe:** Do you think Jacaranda Health should continue to utilize the INTERGROWTH-21st tools? Why or why not?

19.	**ASK AT 12 MONTHS ONLY:**<br>If Jacaranda Health continues using the INTERGROWTH-21st tools, do you think the gestational dating scans should remain free? Why or why not?

THANK YOU FOR TAKING THE TIME TO SPEAK WITH ME TODAY AND SHARING YOUR EXPERIENCE. IS THERE ANYTHING ELSE YOU WOULD LIKE TO SHARE WITH ME ABOUT YOUR EXPERIENCE?
