# Supplementary material for: Implementing the INTERGROWTH-21st gestational dating and fetal and newborn growth standards in peri-urban Nairobi, Kenya: Provider experiences, uptake and clinical decision-making
Source: PLoS One. 2019 Mar 8;14(3):e0213388. doi: 10.1371/journal.pone.0213388 (PMC6407840; doi:10.1371/journal.pone.0213388)
Supplement: S3 File — (PDF) [file pone.0213388.s003.pdf]

S3 FILE. PROVIDER SURVEY: IMPLEMENTATION PHASE (8 MONTHS)

SECTION I: BACKGROUND

I would like to begin by asking you a few questions about yourself and your work at Jacaranda Health.

1. What type of provider are you? (Select one)
    a. Physician
    b. Nurse/Midwife
    c. Ultrasonographer
    d. Clinic Manager

2. What is your role in the Intergrowth project? (Select one)
    a. High Risk Physician
    b. Nurse/Midwife: Conducts Gestational Dating Ultrasound
    c. Nurse/Midwife: Conducts Newborn Anthropometry
    d. Ultrasonographer
    e. Clinic Manager

3. How long have you worked at Jacaranda? (Please indicate "years" or "months" after the number)

SECTION II: ATTITUDES

Now I would like to understand your attitudes regarding a series of statements. For each statement, I will ask you which statement best describes your reaction.

Please indicate how you feel about the following statements:

QUESTION	RESPONSE				
	**Strongly Agree**	**Agree**	**Neither Agree nor Disagree**	**Disagree**	**Strongly Disagree**
4. Use of the Intergrowth tools are part of my usual clinical routine					
5. The Intergrowth tools have increased the quality of care that I am able to provide to clients					
6. Integrating the Intergrowth tools into my daily routine has been worth the time, training and any interruptions to my regular clinical practice					
7. Intergrowth tools have improved my ability to monitor fetal and newborn growth					
8. Intergrowth tools have improved my ability to appropriately identify and refer high risk patients					

Thank you for taking the time complete the survey. Your responses are important for us to assess how the Intergrowth tools are being implemented. We appreciate your time and hard work.
