# Supplementary material for: Implementing the INTERGROWTH-21st gestational dating and fetal and newborn growth standards in peri-urban Nairobi, Kenya: Provider experiences, uptake and clinical decision-making
Source: PLoS One. 2019 Mar 8;14(3):e0213388. doi: 10.1371/journal.pone.0213388 (PMC6407840; doi:10.1371/journal.pone.0213388)
Supplement: S4 File — (PDF) [file pone.0213388.s004.pdf]

S4 FILE. CLINICAL CHART DATA FORMS

Data was entered into a database from client clinical charts. Six forms were created to capture data on the following respective forms within a patient's clinical record.

1. Antenatal Care Record
2. Ultrasound Scan Record and Request
3. Newborn Care Record
4. Maternal and Newborn Discharge Record
5. Clinical Indicators Record
6. Referral Record

S4.1 Antenatal Care Record

This instrument was designed to capture all clinical documentation from antenatal care visits of all women during the study period (pre-implementation and implementation phases). The initial antenatal care visit is captured in the profile form and consequent ANC visits are captured in follow-up forms.

Field Name (Variable Code)	Entry Instructions	Enter for the following ANC visit	Coding
Woman's Study ID (wstudyid)	This is a number that is given to you that corresponds specifically to a Jacaranda Health ID.	All	Numerical code
Type of ANC Visit (typeanc)	Check current visits on p. 4 & 5 of the ANC record to determine type of visit. Confirm by checking billing slip attached to back cover of file. Select one. Specific questions will appear based on the type of visit selected.	All	1, Profile (ANC visit 1) | 0, Follow up (ANC visits 2-8)
Women's Date of Birth (wdob_anc)	Enter full date carefully using the calendar function.<br><br>If date missing:<br>1. Check that JID and woman's name match the front cover of the record and enter the woman's date of birth from the front cover.<br>2. If date of birth is not found on the form or the front cover of the chart, please enter January 1, 1917 (01-01-1917).	All	Use the format DD-MM-YYYY
Women's Age (wage)	Enter 99 if age is blank in record. Do not calculate.	All	Numerical: Two digits
Hypertension – Past (hypert)	If blank, indicate "not recorded."	Profile	1, Yes | 0, No | 9, Not recorded

Diabetes – Past (diabetes)	If blank, indicate "not recorded."	Profile	1, Yes | 0, No | 9, Not recorded
Cardiac Disease – Past (cardiac)	If blank, indicate "not recorded."	Profile	1, Yes | 0, No | 9, Not recorded
Renal Disease – Past (renal)	If blank, indicate "not recorded."	Profile	1, Yes | 0, No | 9, Not recorded
Convulsions – Past (convuls)	If blank, indicate "not recorded."	Profile	1, Yes | 0, No | 9, Not recorded
Mental Illness – Past (mental)	If blank, indicate "not recorded."	Profile	1, Yes | 0, No | 9, Not recorded
Liver Disease – Past (liver)	If blank, indicate "not recorded."	Profile	1, Yes | 0, No | 9, Not recorded
Surgeries – Past (surgery)	If blank, indicate "not recorded."	Profile	1, Yes | 0, No | 9, Not recorded
Surgeries – Chest (chestsurgery)	This question comes up if "yes" is indicated for "surgeries - past."<br><br>Please look for "cardiac" and "heart" in the Notes section on page 1 of the Antenatal Care Record to indicate the type of past surgery. If you find "cardiac" or "heart" in the notes, then select "yes." If not, select "no."	Profile	1, Yes | 0, No
Surgeries – Reproductive (repsurgery)	This question comes up if "yes" is indicated for "surgeries - past."<br><br>Please look for "cs" and "caesarean section" in the Notes section on page 1 of the Antenatal Care Record to indicate the type of past surgery. If you find "cs" or "caesarean section" in the notes, then select "yes." If not, select "no."	Profile	1, Yes | 0, No
Surgeries – Abdominal (absurgery)	This question comes up if "yes" is indicated for "surgeries - past."<br><br>Please look for the word "abdominal" in the Notes section on page 1 of the Antenatal Care Record to indicate the type of past surgery. If you find "abdominal" in the notes, then select "yes." If not, select "no."	Profile	1, Yes | 0, No
Sexually Transmitted Disease – Past (std)	If blank, indicate "not recorded."	Profile	1, Yes | 0, No | 9, Not recorded

Sexually Transmitted Disease – HIV<br>(hivstd)	This question comes up if "yes" is indicated for "sexually transmitted disease - past."<br><br>Please look for the word "HIV" in the Notes section on page 1 of the Antenatal Care Record to indicate the type of past sexually transmitted disease. If you find "HIV" in the notes, then select "yes." If not, select “no.”	Profile	1, Yes | 0, No
Sexually Transmitted Disease – Syphilis<br>(syphstd)	This question comes up if "yes" is indicated for "sexually transmitted disease - past."<br><br>Please look for the word "syphilis" in the Notes section on page 1 of the Antenatal Care Record to indicate the type of past sexually transmitted disease. If you find "syphilis" in the notes, then select "yes." If not, select “no.”	Profile	1, Yes | 0, No
Sexually Transmitted Disease – Gonorrhea<br>(gonstd)	This question comes up if "yes" is indicated for "sexually transmitted disease - past."<br><br>Please look for the word "gonorrhea" in the Notes section on page 1 of the Antenatal Care Record to indicate the type of past sexually transmitted disease. If you find "gonorrhea" in the notes, then select "yes." If not, select “no.”	Profile	1, Yes | 0, No
Sexually Transmitted Disease – HPV<br>(hpvstd)	This question comes up if "yes" is indicated for "sexually transmitted disease - past."<br><br>Please look for the word "HPV" in the Notes section on page 1 of the Antenatal Care Record to indicate the type of past sexually transmitted disease. If you find "HPV" in the notes, then select "yes." If not, select “no.”	Profile	1, Yes | 0, No
Sexually Transmitted Disease - Pelvic Inflammatory Disease (PID)<br>(pidstd)	This question comes up if "yes" is indicated for "sexually transmitted disease - past."<br><br>Please look for the word "PID" or “Pelvic Inflammatory Disease” in the Notes section on page 1 of the	Profile	1, Yes | 0, No

	Antenatal Care Record to indicate the type of past sexually transmitted disease. If you find "PID" or “Pelvic Inflammatory Disease” in the notes, then select "yes." If not, select “no.”		
Asthma – Past / Attacks of Breathlessness<br>(asthma)	Look for either "asthma-past" (new form) or "attacks of breathlessness" (old form)<br>If this variable (in either form) was not indicated on the version of the Antenatal Care Form that you are entering data from (older version), then indicate “Field not available.”<br><br>If blank, indicate “not recorded.”	Profile	1, Yes | 0, No | 9, Not recorded | 8, Field not available
Physical Disability – Past<br>(physdis)	If this variable was not indicated on the version of the Antenatal Care Form that you are entering data from (older version), then indicate “Field not available.”<br><br>If blank, indicate “not recorded.”	Profile	1, Yes | 0, No | 9, Not recorded | 8, Field not available
Endocrine Disorder – Past<br>(endocrine)	If this variable was not indicated on the version of the Antenatal Care Form that you are entering data from (older version), then indicate “Field not available.”<br><br>If blank, indicate “not recorded.”	Profile	1, Yes | 0, No | 9, Not recorded | 8, Field not available
LMP Date COMPLETE – Current Pregnancy<br>(lmpdate_complete_anc)	NEW ANC Record: Page 1<br>OLD ANC Record: Quarter page stapled to the inside cover of the patient file.<br><br>Look at the field for LMP Date on the form and indicate whether:<br>1. A complete date is recorded in the form of Day, Month & Year (preferred always)<br>2. A partial date is recorded in the form of Month & Year only<br>The field is blank / no date is recorded	Profile	1, Date, Month & Year Recorded | 0, Month & Year Only Recorded | 9, No Date Recorded (Blank)
LMP Date – Current Pregnancy<br>(lmpdate_anc_dmy)	NEW ANC Record: Page 1<br>OLD ANC Record: Quarter page stapled to the inside cover of the patient file.	Profile	Use the format DD-MM-YYYY

	Use the calendar function to enter.<br><br>This is linked to your answer for the LMP Date complete variable. Please enter a full date using the calendar.		
LMP Date – Current Pregnancy (lmpdate_anc_my)	NEW ANC Record: Page 1<br>OLD ANC Record: Quarter page stapled to the inside cover of the patient file.<br><br>This is linked to your answer for the LMP Date complete variable. Please enter a month and year in the form of MMYYYY with no spaces (e.g. September 2017 would be 072017).	Profile	Use the format MMYYYY
LMP EDD COMPLETE – Current Pregnancy (lmpedd_complete_anc)	NEW ANC Record: Page 1<br>OLD ANC Record: Quarter page stapled to the inside cover of the patient file.<br><br>Look at the field for LMP EDD on the form and indicate whether:<br>1. A complete date is recorded in the form of Day, Month & Year (always preferred)<br>2. A partial date is recorded in the form of Month & Year only<br>3. The field is blank / no date is recorded	Profile	1, Date, Month & Year Recorded | 0, Month & Year Only Recorded | 9, No Date Recorded (Blank)
LMP EDD - Current Pregnancy (day, month, year) (lmpedd_anc_dmy)	NEW ANC Record: Page 1<br>OLD ANC Record: Quarter page stapled to the inside cover of the patient file.<br><br>Use the calendar function to enter.<br><br>This is linked to your answer for the LMP EDD complete variable. Please enter a full date using the calendar.	Profile	Use the format DD-MM-YYYY
LMP EDD - Current Pregnancy (month, year) (lmpedd_anc_my)	NEW ANC Record: Page 1<br>OLD ANC Record: Quarter page stapled to the inside cover of the patient file.<br>This is linked to your answer for the LMP EDD complete variable. Please enter a month and year in the form of MMYYYY with no spaces (e.g. September 2017 would be 072017).	Profile	Use the format MMYYYY

LMP Certainty - Current Pregnancy (lmpcertain_anc)	NEW ANC Record: Page 1<br>OLD ANC Record: Quarter page stapled to the inside cover of the patient file.<br>If this variable was not indicated on the version of the Antenatal Care Form that you are entering data from (older version), then indicate "Field not available."<br><br>If blank, indicate "not recorded."	Profile	1, Yes | 0, No | 9, Not recorded | 8, Field not available
Gestational Dating Ultrasound EDD (gdus_anc_present)	Indicate if gestational dating ultrasound EDD is included as a field on the form being entered or on the attached sheet on the inside cover of the client file.	All	1, Yes | 0, No
Gestational Dating Ultrasound EDD - Current Pregnancy (gdus_anc)	NEW ANC Record: Page 1<br>OLD ANC Record: Quarter page stapled to the inside cover of the patient file.<br><br>Use the calendar function to enter.<br><br>This variable will only pop up if this field is included on the form being entered.<br><br>If date missing, please enter January 1, 1917 (01-01-1917).	All	Use the format DD-MM-YYYY
Final EDD - Current Pregnancy (Present) (finaledd_preg_present)	Indicate if Final EDD – Current Pregnancy is included as a field on the form being entered.		1, Yes | 0, No
Final EDD - Current Pregnancy (finaledd_preg)	NEW ANC Record: Page 1<br>OLD ANC Record: Quarter page stapled to the inside cover of the patient file.<br><br>Use the calendar function to enter.<br><br>This variable will only pop up if this field is included on the form being entered.<br><br>If date missing, please enter January 1, 1917 (01-01-1917).	All	Use the format DD-MM-YYYY
Gravida (gravida)	If blank, enter 99.	Profile	Numerical

Parity<br>(parity)	If blank, enter 99.<br><br>If there are two numbers with "+" in between, then enter ONLY the number BEFORE the "+" (e.g. "1+1" should be recorded as "1")	Profile	Numerical
In-Vitro Fertilization<br>(ivf)	If this variable was not indicated on the version of the Antenatal Care Form that you are entering data from (older version), then indicate "Field not available."<br><br>If blank, indicate "not recorded."	Profile	1, Yes | 0, No | 9, Not recorded | 8, Field not available
Current Use of Tobacco<br>(tobacco)	If blank, indicate "Not recorded."	Profile	1, Yes | 0, No | 9, Not recorded
Current Use of Alcohol<br>(alcohol)	If blank, indicate "Not recorded."	Profile	1, Yes | 0, No | 9, Not recorded
Family History of Multiple Pregnancies<br>(multpreg)	Please indicate "Yes" or "No" only. There is no need to include additional details regarding the type of family member.<br><br>If blank, indicate "Not recorded."	Profile	1, Yes | 0, No | 9, Not recorded
Miscarriages >1<br>(miscarriage)	If this variable was not indicated on the version of the Antenatal Care Form that you are entering data from (older version), then indicate "Field not available."<br><br>If blank, indicate "not recorded."	Profile	1, Yes | 0, No | 9, Not recorded | 8, Field not available
Evidence of Domestic Abuse<br>(domabuse)	If blank, indicate "Not recorded."	Profile	1, Yes | 0, No | 9, Not recorded
First Visit - Height (cm)<br>(wheight)	Please enter the height in CENTIMETERS.<br><br>If blank, enter 99.9	Profile	Numerical
First Visit – Weight<br>(wweight)	Enter in whatever unit the weight is indicated in the chart – grams or kilograms. Please do not convert.<br><br>If blank, enter 9999	Profile	Numerical

<table>
<tr><td></td><td></td><td></td><td></td></tr>
<tr><td>First Trimester – BMI
(wbmi)</td><td>If blank, indicate “Not recorded.”</td><td>Profile</td><td>3, Underweight (BMI < 18.5) | 2, Normal (BMI 18.5 - 24.9) | 1, Overweight (BMI 25 - 29.9) | 0, Obese (BMI > 30) | 9, Not recorded</td></tr>
<tr><td>Pelvic mass
(pmass)</td><td>If this variable was not indicated on the version of the Antenatal Care Form that you are entering data from (older version), then indicate “Field not available.”

If blank, indicate “not recorded.”</td><td>Profile</td><td>1, Yes | 0, No | 9, Not recorded | 8, Field not available</td></tr>
<tr><td>Laboratory Test Already Entered on ANC PROFILE Form
(labtest_profile)</td><td>When entering data for the ANC follow up visit, you will be asked to check to see if lab test results for rhesus, HIV (maternal) and HIV (paternal) have been entered in the ANC PROFILE. If yes, select “yes.” If no, select “no” and you will then need to enter the values if available. This way if labs were by chance taken after the first visit, we won’t miss them. If data was only recorded for 1-2 of the 3 values, then check the profile form against the updated lab record in the ANC form and if there are additions that have been made at the follow up visit, enter “no” and you will be asked to re-enter the new results of the labs.</td><td>Follow up</td><td>1, Yes | 0, No</td></tr>
<tr><td>Rhesus Status
(rhesus)</td><td>Check the patient’s chart and indicate the results for the lab tests for rhesus and HIV (maternal and paternal). This should be checked at every ANC follow up visit to ensure that this information is captured in case labs were not done during the profile (1st) visit which is usually the case. If blank, indicate “Not recorded.”</td><td>Profile
BUT check at Follow-Up</td><td>1, Positive | 0, Negative | 9, Not recorded</td></tr>
<tr><td>HIV (Maternal)
(hiv_maternal)</td><td>Check the patient’s chart and indicate the results for the lab tests for rhesus and HIV (maternal and paternal). This should be checked at every ANC follow up visit to ensure that this information is captured in case labs were not done during the profile (1st) visit which is usually the case. If blank, indicate “Not recorded.”</td><td>Profile
BUT check at Follow-Up</td><td>1, Reactive | 0, Non-Reactive | 9, Not Recorded</td></tr>
</table>

HIV (Paternal)<br>(hiv_paternal)	Check the patient's chart and indicate the results for the lab tests for rhesus and HIV (maternal and paternal). This should be checked at every ANC follow up visit to ensure that this information is captured in case labs were not done during the profile (1st) visit which is usually the case. If blank, indicate "Not recorded."	Profile<br>BUT check at Follow-Up	1, Reactive | 0, Non-Reactive | 9, Not Recorded
Baby 1-8<br>(baby1, baby2, baby3, …, baby8)	Enter "yes" if row (1-8 respectively) of the table indicated is filled in. If not, enter "no." You will be prompted to fill in information for one baby at a time until you have completed entry for all babies (up to 8 babies if available).	Profile	1, Yes | 0, No
Baby 1-8 - Type of Delivery<br>(typedeliv1, typedeliv2, …, typedeliv8)	If blank, indicate "Not recorded."	Profile	2, Spontaneous Vaginal Delivery (SVD) | 1, Assisted / Vacuum Delivery | 0, Caesarean Section | 9, Not recorded
Baby 1-8 - Birthweight (grams)<br>(bwt1, bwt2, …, bwt8)	Enter in whatever unit the weight is indicated in the chart – grams or kilograms. Please do not convert.<br><br>If blank, enter 9999.	Profile	Numerical
Baby 1-8 - Outcome / Complications<br>(boutcome1, boutcome2, …, boutcome8)	Check all that apply.<br><br>If none of the options are indicated, please check "None of those below."	Profile	8, None of those below | 7, RH isoimmunization (RH) | 6, Multiple Gestation (MG) | 13, Live Female Infant (LFI) | 12, Live Male Infant (LMI) | 11, Alive and Well (A/W) | 5, Living Baby (LB) | 4, Preterm Birth (PB) | 3, Neonatal Loss (NL) | 2, Stillbirth (SB) | 10, Intrauterine Fetal Demise (IUFD) | 1, Miscarriage (MC) | 0, Termination of Pregnancy (TOP)
More than 8 babies<br>(baby_more)	Enter "yes" if there are more than 8 babies / more than 8 rows filled. If not, enter "no."	Profile	1, Yes | 0, No
NOTIFY SUPERVISOR<br>(baby_morenotify)	Let your supervisor know if the patient has more than 8 babies in the past. This is very unlikely.	Profile	

Current ANC Visit – Date<br>(canc_date)	Use the calendar function.<br><br>If date missing, please enter January 1, 1917 (01-01-1917).	All	Use the format DD-MM-YYYY
Current ANC Visit -<br>Gestation (Weeks)<br>(canc_gestwk)	If blank, enter 99.	All	Numerical: Two digits
Current ANC Visit -<br>Gestation (Days)<br>(canc_gestday)	If days are not recorded or unknown, enter 0.<br><br>For example is “x+” is written but no value is provided for days, then indicate “0.”	All	Numerical: 0-6
Current ANC Visit – Blood Pressue (Systolic)<br>(sbp)	Look at the entry in the blood pressure column. It should be indicated as one number over another.<br><br>Please enter the TOP number here.<br><br>This number should be the larger of the two. If it is not (unlikely), then enter the larger number here and indicate this in the comments section at the end of the form.<br><br>If blank, please enter 999.	All	Numerical
Current ANC Visit – Blood Pressue (Diastolic)<br>(dbp)	Look at the entry in the blood pressure column. It should be indicated as one number over another.<br><br>Please enter the BOTTOM number here.<br><br>This number should be the smaller of the two. If it is not (unlikely), then enter the smaller number here and indicate this in the comments section at the end of the form.<br><br>If blank, please enter 999.	All	Numerical
Current ANC Visit -<br>Hb/Anaemia<br>(hemog)	If blank, please enter 99.9.	All	Numerical: One decimal place
Current ANC Visit -<br>Presentation "Breech"<br>(breech)	Look to see if the keyword “breech” is mentioned.	All	1, "Breech" mentioned | 0, "Breech" not mentioned

Current ANC Visit - Weight Gain (format) (wtgain_format)	Please indicate whether a new weight is indicated or if the actual change in weight is indicated. This will allow you to then enter the appropriate weight or weight gain.<br><br>In the new form, this can be found in the current ANC visits table on page 4 & 5. In the old form, it can be found at the bottom of page 5.<br><br>Steps to making this decision:<br>1. Look to see if weight gain is filled.<br>2. If yes, look to see if the number entered is the weight gained (smaller number < 16kg) or the current weight (larger weight >40kg).<br>3. Select weight gain or current weight based on this. If the field is blank, enter "not recorded."	All	1, Current weight indicated | 0, Weight gain indicated (change in weight) | 9, Not recorded
Current ANC Visit – Current Weight (kg) (currentwt)	Enter in KILOGRAMS the current weight. Please do not calculate the difference.<br>In the new form, this can be found in the current ANC visits table on page 4&5. In the old form, it can be found at the bottom of page 5.	Follow-up	Numerical: one decimal place
Current ANC Visit - Weight Gain (kg) (wtgain)	Enter in KILOGRAMS the weight gain (change in weight).<br>In the new form, this can be found in the current ANC visits table on page 4&5. In the old form, it can be found at the bottom of page 5.	Follow-up	Numerical: one decimal place
Current ANC Visit - Vaginal Discharge Color (vagdiscolor)	Look to see if the keyword "red" is mentioned. If this field is not available on the version of the form, please select "field not available."<br><br>o If the column indicating vaginal discharge details IS NOT on the paper form → "field not available"<br>o If the column indicating vaginal discharge details IS on the paper form AND is BLANK → "not recorded"<br>o If the column indicating vaginal discharge details IS on the paper	All	1, Red mentioned | 0, Red NOT mentioned | 8, Field not available| 9, Not recorded

	form AND the word “red” is mentioned → “red mentioned”<br>o If the column indicating vaginal discharge details IS on the paper form AND the word “red” is NOT mentioned (perhaps a different color or some other word is mentioned) → “red not mentioned”		
Current ANC Visit - Comments & Plan for Care (hrnotes)	In the comments & plan for care section, look for the keywords: "vaginal bleeding," "high risk" and "referral." Check all that apply.	All	3, Gyn review | 2, Vaginal bleeding | 1, High risk | 0, Referral | 9, None of the above
Current ANC Visit – Referral (referral_anc)	In the referral column, look for the keywords: "high risk", "tertiary", "external", "internal" and "referral." Check all that apply.	All	4, High risk | 3, Referral | 2, Tertiary | 1, Internal | 0, External | 9, None of the above
Information Recorded for Gestational Dating Ultrasound (gestus)	1. **Check the form to see if this section is available.**<br>If not, mark "fields not available."<br><br>2. **If these fields are available on the forms**, check whether the information is filled.<br>If not, mark "no."<br><br>3. **If these fields are filled**, please do the following:<br>A) Check the scan date and compare it with the date of the current visit being entered (e.g. ANC Visit 1, ANC Visit 2 etc.)<br>B) Enter this information on gestational dating ONLY for the current visit for which the dates correspond.<br><br>4. **If this section is filled <u>but</u> the date of the scan does not correspond to the visit being entered**, please fill "no."<br><br>5. **If this section is filled <u>but</u> the date of the scan is NOT available**, select "yes' and enter data in the profile ANC VISIT 1 form ONLY.<br><br>**<u>Additional details:</u>**<br>The fields you are looking for are:<br>- Gestational dating ultrasound-Performed by<br>- Gestational dating ultrasound-LMP	All	1, Yes | 0, No | 8, Fields not available

	date<br>- Gestational dating ultrasound-LMP (certain/uncertain)<br>- Gestational dating ultrasound-Date of scan<br>- Gestational dating ultrasound-EDD from scan<br>- Gestational dating ultrasound-EDD from LMP<br>- Gestational dating ultrasound-Ultrasound completed<br>- Reasons for ultrasound not completed<br>- Final EDD<br>- By (LMP / ultrasound)<br>- At (weeks)<br><br>NEW Form: You will find this information in the first box on p. 6.<br><br>OLD Form: You will sometimes find this information on a quarter sheet attached to the inside cover of the patient record. If no quarter sheet attached, then these fields are not available. Please indicate "Field not available" for the first question in this section (Information Recorded for Gestational Dating Ultrasound).<br><br>Do NOT pull information from a different form/record. In case some fields are present while others are not, indicate "field not available" for those that are not.		
Gestational Dating Ultrasound - Performed By (provider_gestus)	If blank, please indicate “Not recorded.”	All	2, JH sonographer | 1, JH nurse | 0, Other | 9, Not recorded | 8, Field not available
Gestational Dating Ultrasound - LMP Date COMPLETE (lmpdate_complete_gestus)	Look at the field for LMP Date on the form and indicate whether:<br>1. A complete date is recorded in the form of Day, Month & Year<br>2. A partial date is recorded in the form of Month & Year only<br>The field is blank / no date is recorded	All	1, Date, Month & Year Recorded | 0, Month & Year Only Recorded | 9, No Date Recorded (Blank) | 8, Field not available
Gestational Dating Ultrasound - LMP Date (lmpdate_gestus_dmy)	This is linked to your answer for the LMP Date complete variable. Please enter a full date using the calendar.	All	Use the format DD-MM-YYYY

Gestational Dating Ultrasound - LMP Date (lmpdate_gestus_my)	This is linked to your answer for the LMP Date complete variable. Please enter a month and year in the form of MMYYYY with no spaces (e.g. September 2017 would be 072017).	All	Use the format MMYYYY
Gestational Dating Ultrasound - LMP Certainty (lmpcertain_gestus)	If blank, indicate "Not recorded."	All	1, Certain | 0, Uncertain | 9, Not recorded | 8, Field not available
Gestational Dating Ultrasound - Date of Scan (datescan_gestus)	If date missing, please enter January 1, 1917 (01-01-1917).	All	Use the format DD-MM-YYYY
Gestational Dating Ultrasound - EDD from Scan (eddscan_gestus)	If date missing, please enter January 1, 1917 (01-01-1917).	All	Use the format DD-MM-YYYY
Gestational Dating Ultrasound - EDD from LMP COMPLETE (eddlmp_complete_gestus)	Look at the field for EDD for LMP on the form and indicate whether:<br>3. A complete date is recorded in the form of Day, Month & Year<br>4. A partial date is recorded in the form of Month & Year only<br>5. The field is blank / no date is recorded	All	1, Date, Month & Year Recorded | 0, Month & Year Only Recorded | 9, No Date Recorded (Blank) | 8, Field not available
Gestational Dating Ultrasound - EDD from LMP (day, month, year) (eddlmp_gestus_dmy)	This is linked to your answer for the EDD for LMP complete variable. Please enter a full date using the calendar.	All	Use the format DD-MM-YYYY
Gestational Dating Ultrasound - EDD from LMP (month, year) (eddlmp_gestus_my)	This is linked to your answer for the EDD for LMP complete variable. Please enter a month and year in the form of MMYYYY with no spaces (e.g. September 2017 would be 072017).	All	Use the format MMYYYY
Gestational Dating Ultrasound - Ultrasound Completed (uscompleete_gestus)	If blank, indicate "Not recorded."	All	1, Yes | 0, No | 9, Not recorded | 8, Field not available

Reasons for Ultrasound Not Completed (optout_gestus)	Check all that apply<br><br>If blank, indicate "Not recorded."	All	8, Ultrasound completed prior to this visit | 7, Gestation < 8 weeks (too early) | 6, Gestation >26 weeks (too late) | 5, Wait time | 4, Safety | 3, Not important | 2, Plans to come back | 1, Other | 9, Not recorded
Other Reason (optout_other)	Enter the reason as it appears on the form if indicated.	All	Text
Final EDD (finaledd_anc)	If date missing, please enter January 1, 1917 (01-01-1917).	All	Use the format DD-MM-YYYY
By (byfinaledd_anc)	If blank, indicate "Not recorded."	All	1, By LMP | 0, By ultrasound | 9, Not recorded |8, Field not available
At weeks (wkfinaledd_anc)	If blank, enter 99.<br>If not available, enter 88	All	Numerical: two digits
At days (dayfinaledd_anc)	If days are not recorded or unknown, enter 0.<br><br>For example is "x+" is written but no value is provided for days, then indicate "0."	All	Numerical: 0-6
ANTD1 (at or around 28 weeks) - Current Dose - Date Given (antd1date)	If date missing, please enter January 1, 1917 (01/01/1917)	All	Use the format DD-MM-YYYY
ANTD1 (at or around 28 weeks) - Current Dose – Gestation (antd1gest)	If blank, enter 99.	All	Numerical: two digits
DATA ENTRY COMMENTS (comments_anc)	This is not a required field. Please use it to enter any comments regarding the form that was entered that you would like to share with your supervisor.	All	Text

S4.2 Ultrasound Scan Request & Record

This instrument was designed to capture the details of all ultrasound scans administered to a pregnant woman at Jacaranda Health during the study period.

Field Name<br>(Variable code)	Instructions	Coding
Woman's Study ID<br>(wstudyid)	This is a number that is given to you that corresponds specifically to a JID. It is important that it is entered carefully.	Numerical code
Type of Examination<br>(examtype_usrr)	Indicate what type of ultrasound is recorded on the form.<br><br>If blank, enter “Not recorded.”	0, Obstetric | 1, Pelvis | 2, Breast | 3, Abdomen/Renal/Liver | 4, Thyroid | 5, Prostate/Scrotum | 6, Other | 9, Not recorded
Gestational Dating Ultrasound Performed?<br>(gd_usrr)	If this field is not on the charting form, select "Not available"	1, Yes | 2, No | 8, Not available (field not included in form) | 9, Not recorded (field is blank on form)
Number of Babies<br>(fetus_usrr)	Please look at the Biometrics table and indicate whether information is entered for 1 or 2 babies. If for 2 babies, there will be two sets of results for each parameter.	1, One baby| 0, Two babies | 9, Not recorded
Fetus A - CRL (yes/no)<br>Fetus B - CRL (yes/no)<br>(crlapresent_usrr;<br>crlbpresent_usrr)	Indicate if this field is filled on the form with a "yes" or "no." The units can vary from weeks/days to centimeters/millimeters to percentiles. The only thing we want to know is whether a measurement was done and some result (in any units) was recorded.	1, Yes | 0, No
Fetus A - HC (yes/no)<br>Fetus B - HC (yes/no)<br>(hcapresent_usrr;<br>hcbpresent_usrr)	Indicate if this field is filled on the form with a "yes" or "no." The units can vary from weeks/days to centimeters/millimeters to percentiles. The only thing we want to know is whether a measurement was done and some result (in any units) was recorded.	1, Yes | 0, No
Fetus A - FL (yes/no)<br>Fetus B - FL (yes/no)<br>(flapresent_usrr;<br>flbpresent_usrr)	Indicate if this field is filled on the form with a "yes" or "no." The units can vary from weeks/days to centimeters/millimeters to percentiles. The only thing we want to know is whether a measurement was done and some result (in any units) was recorded.	1, Yes | 0, No
Fetus A - BPD (yes/no)<br>Fetus B - BPD (yes/no)<br>(bpdapresent_usrr;<br>bpdbpresent_usrr)	Indicate if this field is filled on the form with a "yes" or "no." The units can vary from weeks/days to centimeters/millimeters to percentiles. The only thing we want to know is whether a measurement was done and some result (in any units) was recorded. "	1, Yes | 0, No

Fetus A - AC (yes/no)<br>Fetus B - AC (yes/no)<br>(acapresent_usrr;<br>acbpresent_usrr)	Indicate if this field is filled on the form with a "yes" or "no." The units can vary from weeks/days to centimeters/millimeters to percentiles. The only thing we want to know is whether a measurement was done and some result (in any units) was recorded.	1, Yes | 0, No
Fetus A - OFD (yes/no)<br>Fetus B - OFD (yes/no)<br>(ofdapresent_usrr;<br>ofdbpresent_usrr)	Indicate if this field is filled on the form with a "yes" or "no." The units can vary from weeks/days to centimeters/millimeters to percentiles. The only thing we want to know is whether a measurement was done and some result (in any units) was recorded.<br><br>If using an old form and this field is not on that form at all, then enter "Field not available"	1, Yes | 0, No | 8, Field not available
Are the following terms or headings in the record under the Biometrics table?<br>- LMP<br>- LMP gestational age<br>- LMP EDD<br>- Ultrasound gestational age<br>- Ultrasound EDD<br>(new_usrr)	Please select "yes" if you see the following terms/headings listed on the form under the Biometrics table.<br><br>These are only available on the newer forms so will allow us to distinguish between the forms and determine whether it is possible to enter further variables.	1, Yes | 0, No
LMP COMPLETE<br>(lmpbottom_complete_usrr)	Look at the field for LMP on the form and indicate whether:<br>1. A complete date is recorded in the form of Day, Month & Year (this is preferred)<br>2. A partial date is recorded in the form of Month & Year only<br>3. The field is blank / no date is recorded	1, Date, Month & Year Recorded | 0, Month & Year Only Recorded | 9, No Date Recorded (Blank)
LMP<br>(lmpbottom_usrr_dmy)	Enter the LMP towards the bottom left of the Ultrasound Scan Request and Report (under the Biometrics table).<br><br>Use the calendar function.	Use the format<br>DD-MM-YYYY
LMP<br>(lmpbottom_usrr_my)	This is linked to your answer for the LMP complete variable. Please enter a month and year in the form of MMYYYY with no spaces (e.g. September 2017 would be 072017).	Use the format<br>MMYYYY
LMP Gestational Age – Weeks<br>(lmpgestwk_usrr)	If blank, enter 99.	Numerical: Two digits
LMP Gestational Age – Days<br>(lmpgestday_usrr)	If days are not recorded or unknown, enter 0.	Numerical: 0-6
LMP EDD COMPLETE<br>(lmpedd_complete_usrr)	Look at the field for LMP EDD on the form and indicate whether:	1, Date, Month & Year Recorded | 0, Month & Year Only

	1. A complete date is recorded in the form of Day, Month & Year (this is preferred)<br>2. A partial date is recorded in the form of Month & Year only<br>3. The field is blank / no date is recorded<br><br>Use the calendar function.	Recorded | 9, No Date Recorded (Blank)
LMP EDD (day, month, year) (lmpedd_usrr_dmy)	This is linked to your answer for the LMP EDD complete variable. Please enter a full date using the calendar.	Use the format DD-MM-YYYY
LMP EDD (month, year) (lmpedd_usrr_my)	This is linked to your answer for the LMP EDD complete variable. Please enter a month and year in the form of MMYYYY with no spaces (e.g. September 2017 would be 072017).	Use the format MMYYYY
Ultrasound Gestational Age – Weeks (usgestwk_usrr)	If blank, enter 99.	Numerical: Two digits
Ultrasound Gestational Age – Days (usgestday_usrr)	If days are not recorded or unknown, enter 0.	Numerical: 0-6
Ultrasound EDD (usedd_usrr)	If date missing, please enter January 1, 1917 (01/01/1917)<br><br>Use the calendar function.	Use the format DD-MM-YYYY
Check any of the keywords that are mentioned in the REPORT (ALL SCANS) section at the bottom of the form. (report_usrr)	Check all of the following keywords that are mentioned in the Report (All Scans) section.	9, None mentioned | 8, IUGR | 7, Missed miscarriage | 6, Inevitable miscarriage | 5, Molar pregnancy | 4, Ectopic pregnancy | 3, Intrauterine gestational sac | 2, No yolk sac | 1, No fetal pole | 0, Blighted ovum
Ultrasonographer Name (provider_usrr)	Name of provider. Please enter in CAPLOCKS exactly as indicated on the form.<br><br>If the name is not included, please enter "Missing." If the name is not legible, please note and contact your supervisor to help you identify the name of the provider.	Text
Date (date_usrr)	If date missing, please enter January 1, 1917 (01/01/1917)<br><br>Use the calendar function.	Use the format DD-MM-YYYY
DATA ENTRY COMMENTS (comments_usrr)	This is not a required field. Please use it to enter any comments regarding the form that was entered that you would like to share with your supervisor.	All

S4.3 Newborn Admission Record

This instrument was designed to capture all relevant clinical information for every newborn born in the study period. If a woman has twins, her patient chart will have two Newborn Care Records – one for each of the babies.

Field Name<br>(Variable code)	Instructions	Coding
Woman's Study ID<br>(wstudyid)	This is a number that is given to you that corresponds specifically to a JID. It is important that it is entered carefully.	Numerical code
Date of Birth - Newborn 1<br>Date of Birth - Newborn 2<br>(ndob1; ndob2)	If date missing, enter the date of delivery from a separate from. If that is also missing, please enter January 1, 1917 (01/01/1917)	Use the format DD-MM-YYYY
Sex - Newborn 1<br>Sex - Newborn 2<br>(nsex1; nsex2)	If blank, indicate "Not recorded."	2, Male | 1, Female | 0, Indeterminate | 9, Not recorded
EDD COMPLETE – Newborn 1<br>EDD COMPLETE – Newborn 2<br>(nedd_complete1; nedd_complete2)	Look at the field for EDD on the form and indicate whether:<br>1. A complete date is recorded in the form of Day, Month & Year<br>2. A partial date is recorded in the form of Month & Year only<br>3. The field is blank / no date is recorded	1, Date, Month & Year Recorded | 0, Month & Year Only Recorded | 9, No Date Recorded (Blank)
EDD – Newborn 1 (day, month, year)<br>EDD – Newborn 2 (day, month, year)<br>(nedd_dmy1; nedd_dmy2)	This is linked to your answer for the EDD complete variable. Please enter a full date using the calendar.	Use the format DD-MM-YYYY
EDD – Newborn 1 (month, year)<br>EDD – Newborn 2 (month, year)<br>(nedd_my1; nedd_my2)	This is linked to your answer for the EDD complete variable. Please enter a month and year in the form of MMYYYY with no spaces (e.g. September 2017 would be 072017).	Use the format MMYYYY
Gestation (Weeks) - Newborn 1<br>Gestation (Weeks) - Newborn 2<br>(ngest_wk1; ngestwk2)	Enter the number of weeks for gestation. If blank, enter 99.	Numerical
Gestation (Days) - Newborn 1<br>Gestation (Days) - Newborn 2<br>(ngestday1; ngestday2)	Enter the number of days for gestation.<br><br>If days are not recorded or unknown, enter 0.	Numerical<br>Range: 0-6
Mode of Delivery - Caesarean Section (CS) – Newborn 1<br>Mode of Delivery - Caesarean Section (CS) – Newborn 2<br>(ncs1; ncs2)	Please indicate whether caesarean section (CS) is indicated as the mode of delivery.<br><br>If blank, enter "Not recorded."	1, Yes (CS mentioned) | 0, No (CS not mentioned) | 9, Not recorded
Outcome - Newborn 1<br>Outcome - Newborn 2	Enter the status of the newborn.	1, Live birth | 0, Stillbirth | 9, Not recorded

(noutcome1; noutcome2)	If blank, enter "Not recorded."	
Weight - Newborn 1<br>Weight - Newborn 2<br>(nweight1; nweight2)	Enter exactly in the units indicated in the chart – grams or kilograms. Please do not convert.<br>If blank, enter 9999.	Numerical
Length (cm) - Newborn 1<br>Length (cm) - Newborn 2<br>(nlength1; nlength2)	Record length in CENTIMETERS.<br>If blank, enter 99.9.	Numerical: 1 decimal place
Head Circumference (cm) - Newborn 1<br>Head Circumference (cm) - Newborn 2<br>(nhc1; nhc2)	Record head circumference in CENTIMETERS.<br><br>If blank, enter 99.9.<br><br>If the field is not available on the form, enter 88.8.	Numerical: 1 decimal place
Diagnosis - Newborn 1<br>Diagnosis - Newborn 2<br>(ndiagnosis1; ndiagnosis2)	If blank, enter "Not recorded."<br><br>If entering an older record, this field may not be available. In this case enter "Field not available."	2, Small for gestational age | 1, Appropriate for gestational age | 0, Large for gestational age | 9, Not recorded | 8, Field not available
BIRTHWEIGHT - Mark on International Standards Chart - Newborn 1<br>BIRTHWEIGHT - Mark on International Standards Chart - Newborn 2<br>(nmarkbwt1; nmarkbwt2)	**PRE-IMPLEMENTATION PHASE:** If entering an older record, this field may not be available. In this case enter **"Field not available."**<br><br>Look at the chart to see if the provider made a mark on it.<br><br>If it is hard to tell whether or not a mark is present or where exactly it is, then please select "Mark is hard to decipher."	1, Yes | 0, No | 9, Mark hard to decipher | 8, Field not available
LENGTH - Mark on International Standards Chart - Newborn 1<br>LENGTH - Mark on International Standards Chart - Newborn 2<br>(nmarklength1; nmarklength2)	**PRE-IMPLEMENTATION PHASE:** If entering an older record, this field may not be available. In this case enter **"Field not available."**<br><br>Look at the chart to see if the provider made a mark on it.<br><br>If it is hard to tell whether or not a mark is present or where exactly it is, then please select "Mark is hard to decipher."	1, Yes | 0, No | 9, Mark hard to decipher | 8, Field not available
HEAD CIRCUMFERENCE - Mark on International Standards Chart - Newborn 1<br>HEAD CIRCUMFERENCE - Mark on International Standards Chart -	**PRE-IMPLEMENTATION PHASE:** If entering an older record, this field may not be available. In this case enter **"Field not available."**	1, Yes | 0, No | 9, Mark hard to decipher | 8, Field not available

Newborn 2<br>(nmarkhc1; nmarkhc2)	Look at the chart to see if the provider made a mark on it.<br><br>If it is hard to tell whether or not a mark is present or where exactly it is, then please select "Mark is hard to decipher."	
Tool Used for Manual Check – Newborn 1<br>Tool Used for Manual Check – Newborn 2<br>(manualcheck_tool1; manualcheck_tool2)	Indicate which tool was used to do the manual check of the diagnosis.<br><br>**<u>For the OLD form (PRE-IMPLEMENTATION PHASE):</u>**<br>Indicate **"Not applicable"** since the charts are not included in the old forms.<br><br>**<u>For the NEW form (charts):</u>**<br>If one of the measures - gender of baby, weight, or gestation - are not available, then indicate "Could not calculate – data missing."	2, Newborn Size Online Tool l | 1, Newborn Standards Chart (33-42+6 weeks) | 0, Very Preterm Newborn Reference Chart (24-32+6 weeks) | 9, Could not calculate - data missing | 8, Not applicable (older form)
MANUAL CHECK Diagnosis - Newborn 1<br>MANUAL CHECK Diagnosis - Newborn 2<br>(manualcheck_diagnosis1; manualcheck_diagnosis2)	Do a check yourself to determine if there is agreement for NEW forms ONLY.<br><br>**<u>For OLD Form (PRE-IMPLEMENTATION PHASE):</u>**<br>Indicate **"Not applicable"** since diagnosis is not included as a field.<br><br>**<u>For NEW Form:</u>**<br>Please use the appropriate gender INTERNATIONAL STANDARDS FOR SIZE chart based on weight, gender and gestation to determine the diagnosis yourself. See instructions above for details. Please record what you identified as the diagnosis based on your own training of using the chart.<br><br>If one of the measures - gender of baby, weight, or gestation - are not available, then indicate "Could not calculate – data missing."	2, Small for gestational age | 1, Appropriate for gestational age | 0, Large for gestational age | 9, Could not calculate - data missing | 8, Not applicable (older form)
MANUAL CHECK Correct Gender Chart Used? - Newborn 1<br>MANUAL CHECK Correct Gender Chart Used? - Newborn 2<br>(ncorrect_chart1; ncorrect_chart2)	**<u>For OLD Form (PRE-IMPLEMENTATION PHASE):</u>**<br>For the older records where the growth charts are not provided, please indicate so by selecting **"Gender charts not part of record (older form)."**<br><br>**<u>For NEW Form:</u>**<br>When doing your own manual calculations, determine whether the provider used the correct gender charts to do their calculations.	1, Yes | 0, No | 8, Gender charts not part of record (older form)

	If not, indicate so with a “no.”	
DATA ENTRY COMMENTS (comments_new1; comments_new2)	This is not a required field. Please use it to enter any comments regarding the form that was entered that you would like to share with your supervisor.	All

S4.4 Maternal and Newborn Discharge Record

This instrument was designed to capture all clinical events that occurred from delivery up to discharge from Jacaranda Health for the mother and her newborn(s).

**Field Name (Variable code)**	**Instructions**	**Coding**
Woman's Study ID (wstudyid)	This is a number that is given to you that corresponds specifically to a JID. It is important that it is entered carefully.	Numerical code
Date of Delivery (datedeliv_dis)	If date missing, enter date of birth from newborn admission record. If no date of birth either, please enter January 1, 1917 (01/01/1917). Please use the calendar function.	Use the format DD-MM-YYYY
Are the following terms or headings in the Maternal and Newborn Discharge Summary?<br>- Caesarean Section<br>- Number of neonates<br>- Complications - Small for Gestational Age<br>- Complications - Large for Gestational Age<br>- Complications - Referral<br>- Complications - Neonatal Death<br>(new_dis)	Please select "yes" if you see the terms / headings listed on the form. This will help identify whether an older or newer version of this form is being entered. These terms are not present on the older version of this form. If one of these terms is located on the form, the others should be as well but please do check that all are included before marking "yes".	1, Yes | 0, No
Caesarean Section (cs_dis)	Select "yes" if the box to the right of "ceserean section" is ticked. If not, check "no". This will only show up if a newer version of the form is being entered.	1, Yes | 0, No
Induction of Labour (induction_dis)	Select "yes" if the box to the right of "induction of labour" is ticked. If not, check "no". If on the old form, neither of two boxes are ticked, select “not recorded.” If field is not on the form, select “field not available.”	1, Yes | 0, No | 9, Not recorded| 8, Field not available
Indication Details (postdate) (postdate)	Select "yes" if the keyword "post-date" is mentioned. If not, select "no."	1, Yes | 0, No
Number of Neonates (neonates_dis)	Indicate the number of babies for each woman. This will only show up if a newer version of the form is being entered.	1, Singleton | 2, Twins | 0, Other

Outcome - Livebirth<br>Outcome - Livebirth (Baby 2)<br>(livebirth_dis; livebirth2_dis)	Data entry options:<br>(1) If old pink form: Look at fetal outcome and indicate "yes" if the text includes the words: "stable", "LMI", "LFI", "Fetal outcome: good", or "alive and well." If unsure, select "field not available."<br>(2) If old white form: Indicate "yes" if the yes box is ticked or "no" if the no box is ticked or "Not recorded" if blank.<br>(3) If newer from: Indicate "yes" if the box to the right of "Livebirth" is ticked. Enter "no" if it is not ticked.<br>Do NOT get this information from another form.	1, Yes | 0, No | 8, Field not available | 9, Not recorded
Complications - Small for Gestational Age<br>Complications - Small for Gestational Age (Baby 2)<br>(sga_dis; sga2_dis)	Enter "yes" if box to the right of "Complications - Small for Gestational Age" is ticked. This will only show up if a newer version of the form is being entered.	1, Yes | 0, No
Complications - Large for Gestational Age<br>Complications - Large for Gestational Age (Baby 2)<br>(lga_dis; lga2_dis)	Enter "yes" if box to the right of "Complications - Large for Gestational Age" is ticked. This will only show up if a newer version of the form is being entered.	1, Yes | 0, No
Complications - Referral<br>Complications - Referral (Baby 2)<br>(referral_dis; referral2_dis)	Enter "yes" if box to the right of "Referral" is ticked. This will only show up if a newer version of the form is being entered.	1, Yes | 0, No
Complications - Referral Indication<br>Complications - Referral Indication (Baby 2)<br>(refindic_dis; refindic2_dis)	Please enter the text written after "Indication"	Text
Complications - Neonatal Death<br>Complications - Neonatal Death (Baby 2)<br>(ndeath_dis; ndeath2_dis)	Enter "yes" if box to the right of "Neonatal Death" is ticked. This will only show up if a newer version of the form is being entered.	1, Yes | 0, No

DATA ENTRY COMMENTS (comments_dis)	This is not a required field. Please use it to enter any comments regarding the form that was entered that you would like to share with your supervisor.	All

S4.5 Clinical Indicators Record

This instrument was designed to capture a small set of specific maternal and neonatal outcome metrics that are tracked by Jacaranda Health management for all deliveries.

Variable Name (Variable code)	Instructions	Coding
Woman's Study ID<br>(wstudyid)	This is a number that is given to you that corresponds specifically to a JID. It is important that it is entered carefully.	Numerical code
Woman's Date of Birth<br>(wdob_clin)	If date missing, please enter January 1, 1917 (01/01/1917)	Use the format DD-MM-YYYY
Type of Delivery - Caesarean Section<br>(cs_clin)	Select "yes" if the box to the left of "Caesarean section" is ticked. If not, check "no".	1, Yes | 0, No
Interventions during Labour / Delivery - Induction of Labour<br>(induction_clin)	Select "yes" if the box to the left of "induction of labour" is ticked. If not, check "no".	1, Yes | 0, No
Indication (postdate)<br>(postdate_clin)	Select "yes" if the keyword "post-date" is mentioned after "indication" for Induction of labour. If not, select "no."	1, Yes | 0, No
Number of Infants Born<br>(neonates_clin)	Indicate which box is checked.	1, Singleton | 2, Twins | 3, Triplets | 0, Other
Newborn Outcome - Livebirth<br>Outcome - Livebirth (Baby 2)<br>(livebirth_clin; livebirth2_clin)	Enter "yes" if box to the left of "Livebirth" is ticked	1, Yes | 0, No
Complications Newborn – Referral<br>Complications Newborn – Referral (Baby 2)<br>(referral_nclin; referral2_nclin)	Enter "yes" if box to the left of "Referral" is ticked. If not enter "no."	1, Yes | 0, No
Complications - Neonatal Death<br>Complications - Neonatal Death (Baby 2)<br>(ndeath_clin; ndeath2_clin)	Enter "yes" if box to the left of "Neonatal Death" is ticked.	1, Yes | 0, No
Date Form Completed<br>(date_clin)	Please use the calendar function. If date missing, please enter January 1, 1917 (01/01/1917).	Use the format DD-MM-YYYY
DATA ENTRY COMMENTS<br>(comments_clin)	This is not a required field. Please use it to enter any comments regarding the form that was entered that you would like to share with your supervisor.	Text

S4.6 Referral Record

This instrument was designed to capture all client (mother and newborn) referrals from Jacaranda Health to higher level facilities.

-

Variable	Instructions	Coding
Woman's Study ID<br>(wstudyid)	This is a number that is given to you that corresponds specifically to a JID. It is important that it is entered carefully.	Numerical code
Type of External Referral<br>(type_ref)	Select one of the options.<br>If blank, select "not recorded."	1, Triage | 2, Inpatient | 3, Outpatient | 9, Not recorded
Time of Referral<br>(time_ref)	Select one of the options.<br>If blank, select "not recorded."	0, Pre-delivery |1, Post-delivery | 9, Not recorded
Type of Patient<br>(patient_ref)	Select one of the options.<br>If blank, select "not recorded."	1, Adult | 0, Pediatric | 9, Not recorded
Reason for Referral<br>(reason_ref)	Please look for the following terms and indicate the one that is mentioned as a reason for referral. If none of these are mentioned, select other. If blank, then select "not recorded."	1, IUGR | 2, Fetal distress / NRFHR | 3, Reduced Fetal Movement | 4, PV bleeding | 5, Preterm labor (PTL) | 6, GDM | 7, Macrosomia | 8, Pre-eclampsia | 9, Placental insufficiency | 10, Prolonged drainage of liquid (prolonged rupture of membranes) | 11, PPROM | 12, PROM | 13, Post-dates | 14, APH | 15, Polyhydramnios | 16, Oligohydramnios | 17, Respiratory distress syndrome | 18, Meconium aspiration syndrome | 19, Other obstetric indications | 20, Other newborn indications | 21, Other maternal indications (non-obstetric) | 99, Not recorded

Date of Referral<br>(date_exoutref)	Please use the calendar function to enter. If date missing, please enter January 1, 1917 (01/01/1917)	Use the format DD-MM-YYYY
DATA ENTRY COMMENTS<br>(comments_ref)	This is not a required field. Please use it to enter any comments regarding the form that was entered that you would like to share with your supervisor.	Text
